# ParSite is a multicolor DNA labeling system that allows for simultaneous imaging of triple genomic loci in living cells

**DOI:** 10.1371/journal.pbio.3003009

**Published:** 2025-01-24

**Authors:** Xiaohui He, Yadong Sun, Hanhui Ma

**Affiliations:** School of Life Science and Technology, ShanghaiTech University, Shanghai, China; National Cancer Institute, UNITED STATES OF AMERICA

## Abstract

The organization of the human genome in space and time is critical for transcriptional regulation and cell fate determination. However, robust methods for tracking genome organization or genomic interactions over time in living cells are lacking. Here, we developed a multicolor DNA labeling system, ParSite, to simultaneously track triple genomic loci in the U2OS cells. The tricolor ParSite system is derived from the *T*. *thermophilus* ParB/ParSc (*Tt*ParB/ParSc) system by rational design. We mutated the interface between *Tt*ParB and ParSc and generated a new pair of *Tt*ParBm and ParSm for genomic DNA labeling. The insertions of 16 base-pair palindromic ParSc and ParSm into genomic loci allow dual-color DNA imaging in living cells. A pair of genomic loci labeled by ParSite could be colocalized with p53-binding protein 1 (53BP1) in response to CRISPR/Cas9-mediated double-strand breaks (DSBs). The ParSite permits tracking promoter and terminator dynamics of the *APP* gene, which spans 290 kilobases in length. Intriguingly, the hybrid ParS (ParSh) of half-ParSc and half-ParSm enables for the visualization of a third locus independent of ParSc or ParSm. We simultaneously labeled 3 loci with a genomic distance of 36, 89, and 352 kilobases downstream the C3 repeat locus, respectively. In sum, the ParSite is a robust DNA labeling system for tracking multiple genomic loci in space and time in living cells.

## Abstract 2

Live cell imaging tools are used to study chromatin dynamics over time, but a simple and versatile multicolor DNA imaging method is still needed. This study develops ParSite, based on the T. thermophilus ParB/ParS system, that allows for tri-color genomic DNA imaging in living cells.

## Introduction

Chromatin structure and dynamics are important for gene regulation and DNA repair [1,2]. For chromatin structure, many methods have been developed to resolve its fine folding in vitro [3] or in vivo [4]. Chromosome conformation capture [5,6] (3C) and its derived Hi-C [7] have been applied for studies of 3D genome organization extensively. Hi-C data can define genomic interactions or loops, topologically associating domains (TADs), and A/B compartments from a population of cells [7–9]. It is challenging to identify genomic interactions or loops by Hi-C in single cells. DNA FISH such as OligoPaint [10], MERFISH [11,12], Hi-M [13], or ORCA [14] allows for direct visualization of chromatin structures from kilobase to megabase scales in cells. These methods were used to identify genome folding in 3D in fixed cells. Nevertheless, the genome structure is highly dynamic over time in living cells. It is critical to have approaches for visualization or tracking genomic interactions and folding in real-time (the fourth dimension).

Live cell imaging tools aid us to study chromatin dynamics. The fluorescence repressor operator system (FROS) [15,16], CRISPR-based imaging system [17–21], or the ParB/ParS [22–24] system allows us to track genomic loci in living cells. The FROS achieves live cell DNA imaging by the integration of tandem lac operator (LacO) sequences and recruiting its binding partner lac repressor fused with fluorescent protein [15]. At least 20 copies of LacO (typically 100 to 300 copies and up to 100,000 copies) are required for successful DNA imaging but with a low signal-to-noise ratio (SNR) [25]. CuO array or TetO [26] array could be also combined with LacO for multicolor DNA imaging but with a requirement for a minimal of 144 [26] or 28 [27] copies, respectively. The DNA repeat fragment clone and integration for large copies of the FROS array are difficult to tackle. Besides, the integration of multiple copies of FROS sequence may also perturb genome structure and function. For minimal copies of FROS array, low SNR constrains its application in live-cell DNA imaging, in certain cell types with cutting-edge microscopes. CRISPR-based DNA imaging was achieved by targeting the tandem repeats in the human genome using nuclease dead Cas9 (dCas9) fused with the fluorescent proteins along with the cognate sgRNA. CRISPRainbow [20] could be used for simultaneous visualization of 6 genomic loci with high copies of tandem repeats, while CRISPR-Sirius [21] permits labeling the tandem repeats as low as 20 copies. Recently, the non-repetitive DNA was visualized by liquid–liquid phase separation (LLPS)-mediated signal amplification on the genomic locus targeted by a single sgRNA [28]. For CRISPR-based DNA imaging systems, the transient transfection of sgRNA and Cas protein causes heterogenous SNR among cells compared to isolated clones from FROS or other stable cell line-based methods. The heterogenous SNR is a long-standing issue for the multicolor DNA imaging field. The CRISPR-based system has achieved repetitive and non-repetitive DNA imaging with success on a case-by-case basis [17–21,28]. The dCas9 binding to genome DNA may also hinder transcription and stall replication initiation or elongation. Although there are some possible methods to achieve dual- or multicolor DNA labeling through a combination of the above tools, however, a simple and versatile multicolor DNA imaging method from one system is still needed. The ParB/ParS-based ANCHOR systems achieve DNA visualization by integrating sequence from bacteria containing ParS into a genomic locus, which allows the fluorescent protein-fused ParB proteins to spread kilobases around ParS [22]. Recent study also shows that ParS and ParB associate to form nanometer-sized spherical condensates in bacteria [29]. We have previously developed mParSpot based on the ParB-ParS and Noc-NBS systems (another component from bacteria) for tracking pairwise genomic loci in living cells [30]; nevertheless, the mParSpot is primarily a dual-color DNA imaging system.

Here, we developed a multicolor ParSite system based on *T*. *thermophilus* ParB/ParS (*Tt*ParB/ParSc) system. By mutation of the interface amino acids between *Tt*ParB and ParSc, we generated a new pair of *Tt*ParBm and ParSm for dual-color DNA imaging. This paper also generated the hybrid of half-ParSc and half-ParSm (ParShybrid, ParSh) which allows for tri-color genomic DNA imaging. Thus, we believe the multicolor ParSite is a robust and sensitive DNA labeling system for exploring genome structure and dynamics in living cells.

## Results

### Rational design of *Tt*ParBm for dual-color DNA labeling

**Fig 1:**
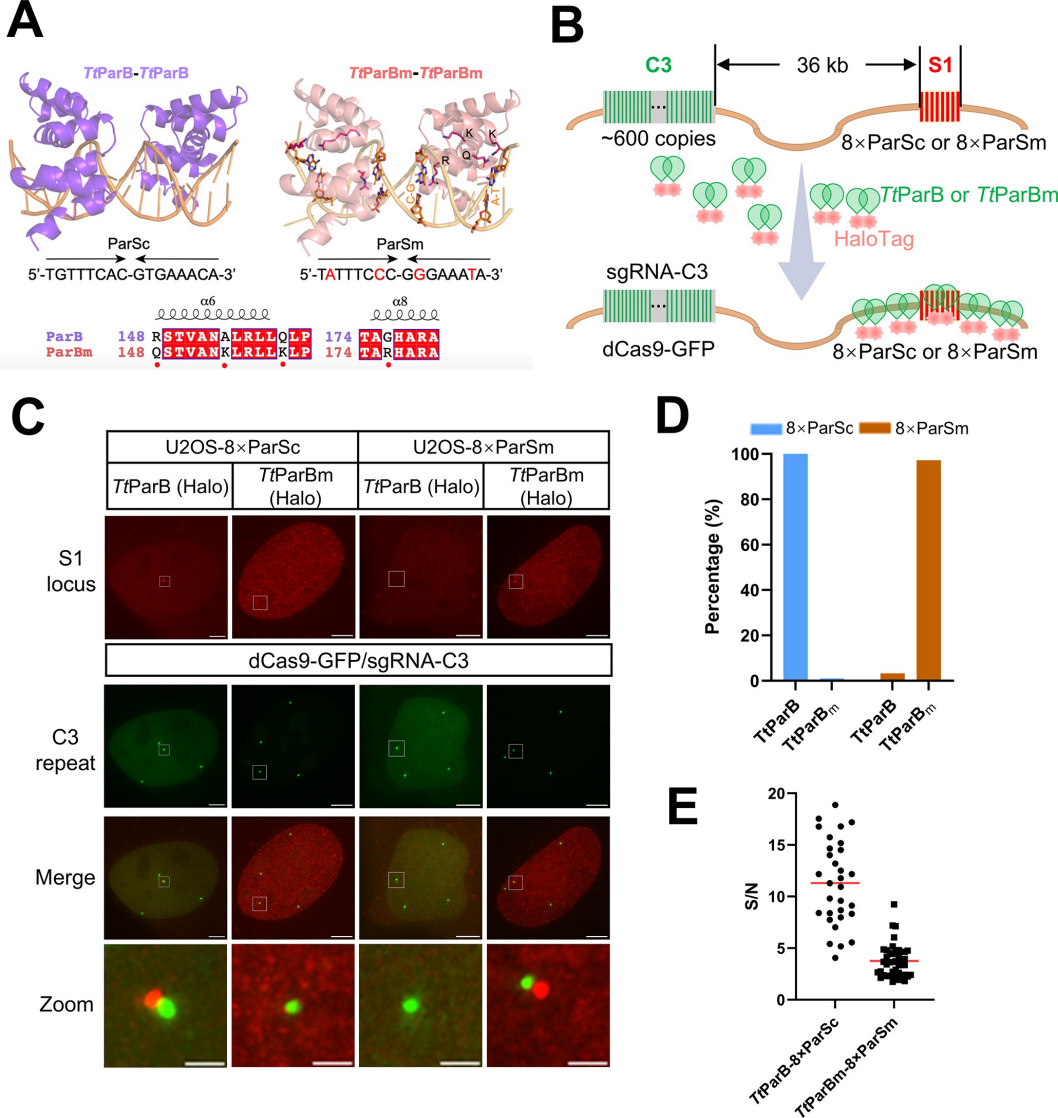
Generation of *Tt*ParB-ParSc mutants for live cell DNA imaging. (A) AlphaFold models of DBDs from dimeric *Tt*ParB (top, purple) and *Tt*ParBm (top, red) binding ParSc and ParSm, respectively. The organizations of *Tt*ParB or *Tt*ParBm and DNA (yellow line) are based on recent structural studies (PDB entry 6T1F and 7OL9). The nucleotide sequences of the ParSc and ParSm are shown below the structure. The red letters indicate the nucleotide differences between ParSc and ParSm. Sequence alignment of α6 and α8 in the DBD of *Tt*ParB and *Tt*ParBm are shown below. Red dots mean the mutation sites and their locations in the 3D structure are plotted. (B) The schematic of *Tt*ParB-8×ParSc or *Tt*ParBm-8×ParSm for DNA imaging; 8×ParSc or 8×ParSm is integrated in S1 locus which is 36 kb downstream of C3 repeat (as an endogenous reference locus). (C) Representative images of U2OS-8×ParSc or U2OS-8×ParSm cell line labeled by *Tt*ParB-HaloTag or *Tt*ParBm-HaloTag, respectively. C3 repeat was labeled by dCas9-GFP/sgRNA-C3. Scale bars, 5 μm for images with the whole cells and 1 μm for zoomed images. (D) Percentage of 8×ParSc (blue) or 8×ParSm (brown) cells labeled by *Tt*ParB-HaloTag or *Tt*ParBm-HaloTag, respectively, *n* = 22, 22, 30, 36 cells for each group from left to right. (E) The signal (S)-to-noise (N) ratio of DNA imaging by *Tt*ParB-8×ParSc or *Tt*ParBm-8×ParSm. The red line indicates the mean value for each group. *n* = 31 for *Tt*ParB-8×ParSc group, 42 for *Tt*ParBm-8×ParSm group. The underlying data associated with this figure are available in S1 Data.

To expand the ParB/ParS-based DNA imaging toolbox in living cells, we rationally designed mutations for ParB protein from *Thermus thermophilus* (**Fig 1A**). As we published previously [30], *Tt*ParB showed the best SNR for genomic loci-specific DNA imaging among orthogonal ParBs and paralogous Nocs. Interestingly DNA-binding specificity of ParBs can be switched from ParS to NBS by mutations of 4 conserved amino acids [31]. Through protein sequence alignment, we mutated these 4 respective positions in *Tt*ParB resulting in *Tt*ParBm (*Tt*ParB mutant) and also mutated 4 nucleotides in ParSc (ParS consensus) toward to NBS-like sequences, named ParSm (ParS mutant) (**Fig 1A**).

To test the labeling efficiency and specificity of *Tt*ParB/ParSc and *Tt*ParBm/ParSm, we integrate 8 copies of ParSc or ParSm into the S1 locus, 36 kb downstream the C3 locus, a repetitive region (approximately 600 copies) in chromosome 3 (**Figs 1B and S1**). After transient transfection of *Tt*ParB or *Tt*ParBm into U2OS-8×ParSc or U2OS-8×ParSm cells, respectively, *Tt*ParB could specifically label S1 locus integrated with 8×ParSc and *Tt*ParBm with 8×ParSm without cross-reaction (**Fig 1C**). As statistical data shown in **Fig 1D**, the labeling efficiency is 100% for *Tt*ParB/8×ParSc and 97.22% for *Tt*ParBm/8×ParSm. The mean value of SNR is 11.31 for *Tt*ParB/8×ParSc and 3.75 for *Tt*ParBm/8×ParSm (**Fig 1E**). To clarify the factors influencing SNR, we quantified the expression level of ParB and ParBm in each cell. The background fluorescence intensity of ParB or ParBm is negatively correlated to the SNR (**S2 Fig**). Thus, *Tt*ParB/8×ParSc and *Tt*ParBm/8×ParSm could be utilized for dual-color DNA imaging and we termed this system as dParSite (dual-color ParSite).

### Simultaneously labeling a pair of genomic loci by dParSite

**Fig 2:**
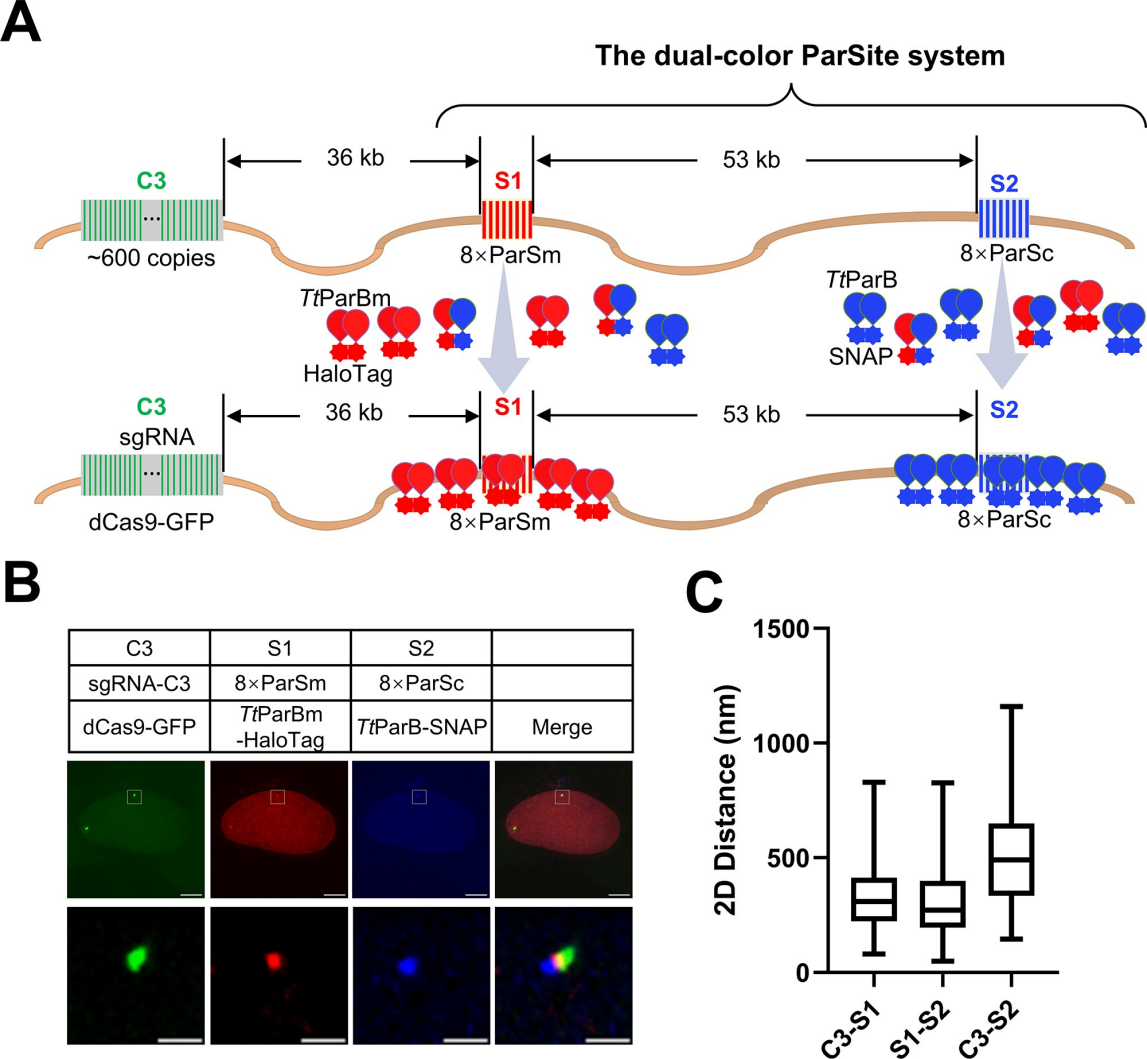
Imaging of pairwise genomic loci by the dual-color ParSite system. (A) The diagram shows the dual-color ParSite system for imaging of pairwise DNA loci. U2OS cells were integrated with 8×ParSm at S1 locus and 8×ParSc at S2 locus resulting in U2OS-8×ParSm-8×ParSc cell lines. *Tt*ParBm-HaloTag and *Tt*ParB-SNAP along with SgRNA-C3 and dCas9-GFP were co-transfected into cells to image the C3, S1, and S2 loci simultaneously. Combining ParB/ParSc and ParBm/ParSm constitutes the dual color ParSite system. (B) Representative images of S1 and S2 locus in A labeled by ParSite system. Scale bars, 5 μm for images with the whole cells and 1 μm for zoomed images. (C) Box-and-Whisker plots of 2D spatial distances among C3-S1, S1-S2, and C3-S2. *n* = 38 for each group. Box spans from second to third quartiles. Whiskers represent min to max value, and middle lines represent the position of the median value of the data distribution. The underlying data associated with this figure are available in S2 Data.

To confirm that the dParSite could simultaneously label 2 target sites on a single chromosome, we knocked in 8×ParSm at the S1 locus, 36 kb downstream of the C3 locus (approximately 600 copies) and 8×ParSc at the S2 locus, 89 kb downstream of the C3 locus into U2OS cells. We transfected *Tt*ParBm-HaloTag to visualize the S1 locus and *Tt*ParB-SNAP to visualize the S2 locus, along with dCas9-GFP/sgRNA-C3 for labeling C3 locus (**Fig 2A**). As shown in **Fig 2B**, the S1 spot (8×ParSm in red) and S2 spot (8×ParSc in blue) adjacent to the C3 spot (C3 repeat in green) were visualized simultaneously in the U2OS-8×ParSm-8×ParSc cells. The genomic distance between C3 and S1, S1 and S2, or C3 and S2 is 36 kb, 53 kb or 89 kb, respectively. As shown in **Fig 2C**, the 2D spatial distances of these loci were measured to range from 80.4 to 829.4 nm with a mean of 332.98 nm for C3 and S1, 48.84 to 826.9 nm with a mean of 308.36 nm for S1 and S2, 145.12 to 1159.88 nm with a mean of 502.39 nm for C3 and S2 from images with 3D projection. We also measured the 3D distance of pairwise genomic loci. It also shows a heterogenous distribution with almost the same pattern as the one from 2D distance (**S3A and S3B Fig**). To verify the correct localization of the ParSite Tag, we used CRISPR-based labeling of C3 as a reference, which is 36 kb upstream of the S1 site. It will be intriguing to further explore whether CRISPR-based labeling or ParSite labeling affects chromatin topology or their spatial distance. The foci areas from 3D projected images also show heterogeneous distribution (**S3C Fig**). The labeling efficiency of dParSite is 90.5% (**S4 Fig**).

### GFP-53BP1 foci concurrently associate with 2 adjacent sites labeled by dParSite

**Fig 3:**
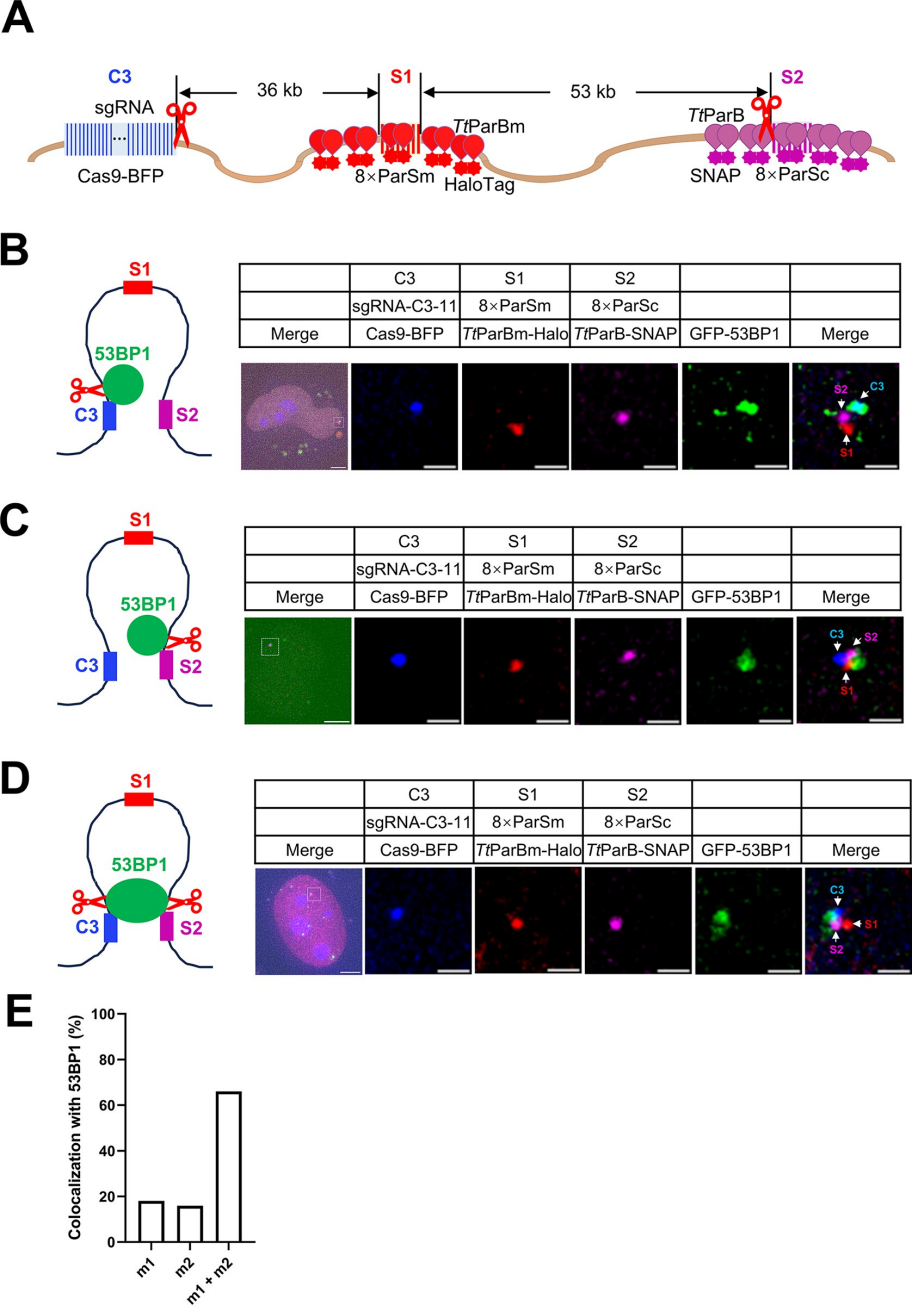
Visualizing the genomic loci with DNA DSBs by the ParSite system in live cells. (A) The schematic of labeling genomic loci with DNA DSBs using the ParSite system. The 2 red scissors represent 2 cleavage sites mediated by the CRISPR-Cas9 system. (B) Colocalization of the CRISPR-mediated cleavage site adjacent to C3 locus and 53BP1. The cleavage site (scissor) adjacent to the C3 repeat was shown in the diagram. GFP-53BP1 was chosen to indicate the DSB induced by the CRISPR-Cas9/sgRNA system. Scale bars, 5 μm for images with the whole cell and 1 μm for zoomed images. (C) Colocalization of the CRISPR-mediated cleavage site adjacent to the S2 locus and 53BP1. The cleavage site (scissor) adjacent to the S2 locus was shown in the diagram. Scale bars, 5 μm for images with the whole cell and 1 μm for zoomed images. (D) Colocalization of the CRISPR-mediated cleavage at 2 DNA sites and 53BP1. The cleavage sites (scissors) with one close to the C3 repeat and the other close to the S2 locus. Scale bars, 5 μm for images with the whole cell and 1 μm for zoomed images. (E) The percentage of cells grouping in B–D. m1 means only C3 locus colocalized with 53BP1 protein and m2 means only S2 locus colocalized with 53BP1 protein. m1 + m2 means both C3 locus and S2 locus colocalized with 53BP1 protein. *n* = 17 cells. The underlying data associated with this figure are available in S3 Data.

53BP1, a key regulator of the DNA repair pathway, forms foci around the double-strand break (DSB) site right after the DNA damage occurs [32]. Here, we introduced DSBs by CRISPR Cas9 nuclease 5 kb downstream of C3 locus and/or 10 kb upstream of S2 locus (**Fig 3A**), and then examined the colocalization between C3 spot or S2 spot and 53BP1 foci. We have previously shown that C3 locus can be visualized by Cas9 nuclease and truncated guide RNA (11 nucleotides in length), which are sufficient to recognize target sites but lacking cleavage activity [33]. As shown in **Fig 3B–3D**, 53BP1 foci were associated with C3 when the cleavage site adjacent to C3 repeat; 53BP1 foci were associated with S2 when the cleavage site adjacent to S2; 53BP1 foci were associated with both C3 and S2 when the cleavages occur at both sites. The percentage of cells having C3 locus colocalized with 53BP1 foci is 18%. The percentage is 15.9% for the S2 locus colocalized with 53BP1 foci. The percentage of cells with both C3 and S2 loci colocalized with 53BP1 foci is 66.03% (**Fig 3E**). When no CRISPR-mediated DNA cleavage is induced, there are no 53BP1 foci colocalized with C3, S1, or S2 loci (**S5 Fig**). These data suggest 53BP1 forms foci around the DSB sites, and two 53BP1 foci may fuse together when two DSBs occur.

### Imaging promoter and terminator of the *APP* gene by the dParSite

**Fig 4:**
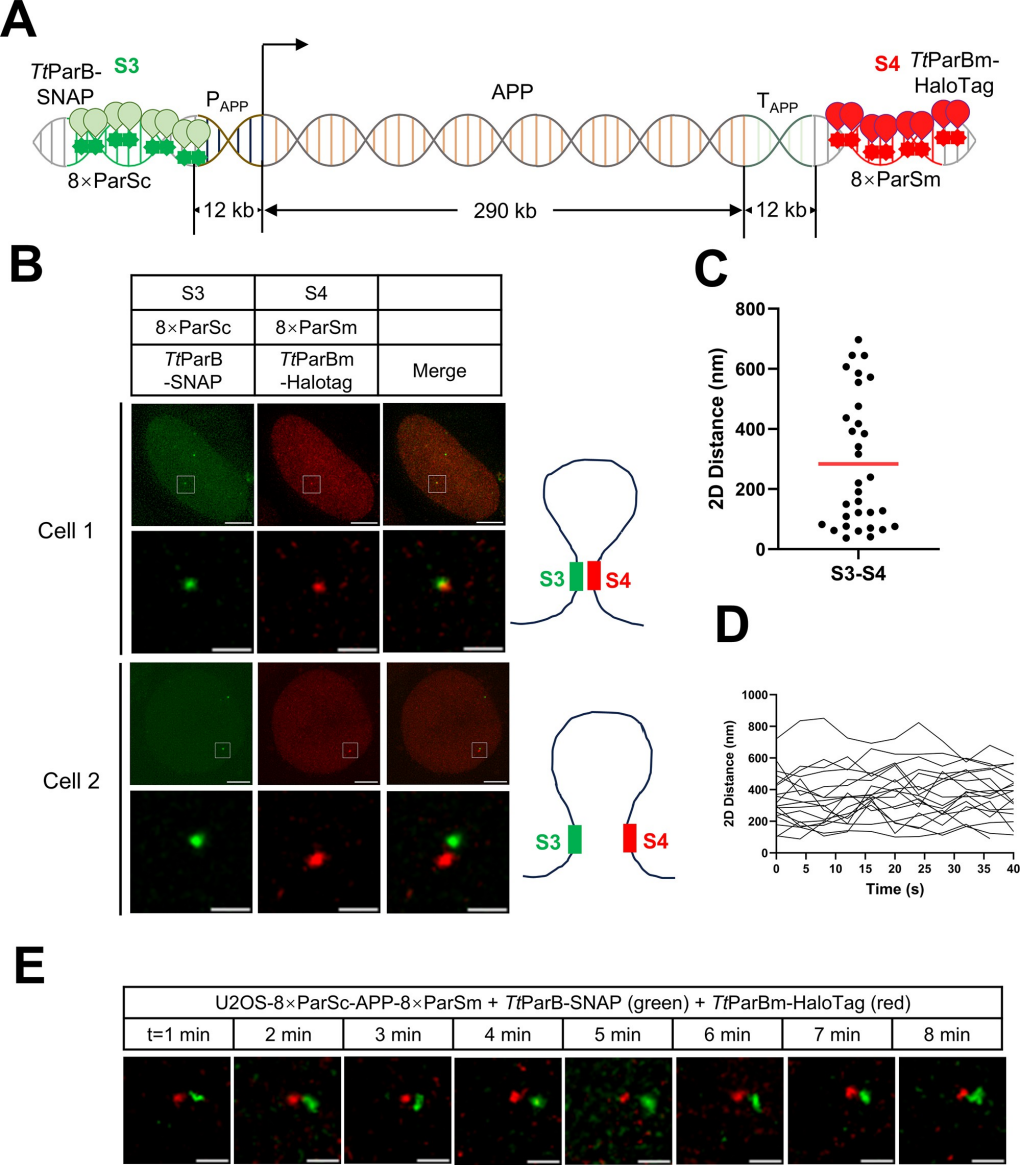
Tracking the dynamics of the promoter and terminator of the *APP* gene by the ParSite system. (A) Schematic of labeling promoter and terminator of the *APP* gene by ParSite. *Tt*ParB-SNAP and *Tt*ParBm-HaloTag were transfected together into cell line U2OS-8×ParSc-APP-8×ParSm to label the loci S3 (promoter region) and S4 (terminator region). P_APP_ is the promoter region of the *APP* gene and T_APP_ is the terminator region of the *APP* gene. (B) Representative images of S3 and S4 loci labeled by the ParSite system. The images of Cell 1 and Cell 2 are captured from the same single-cell derived U2OS-8×ParSc-APP-8×ParSm line. The diagrams on the right indicate the gene loop states. Scale bars, 5 μm for images with the whole cells and 1 μm for zoomed images. (C) Statistics of 2D spatial distance between S3 and S4. The red line indicates the mean. *n* = 32. (D) Live tracking the 2D distances between the promoter and terminator of the *APP* gene. The distances between the promoter (S3) and terminator (S4) of the *APP* gene at each time point over 40 s were measured and plotted. (E) Time-lapse of S3 and S4 loci pair. This pair of loci was tracked over 8 min with a time interval of 1 min. Scale bars, 1 μm. The underlying data associated with this figure are available in S4 Data.

It has been proposed that the promoter and terminator may interact during active transcription [34]. To examine promoter and terminator interactions, we chose the human Amyloid Beta Precursor Protein (*APP*) gene, which spans 290 kb on chromosome 21 and is actively transcribed. To avoid the impact of foreign gene expression on chromatin dynamics, we only inserted 8×ParSc 12 kilobases upstream (S3 locus marked as the promoter region) and 8×ParSm 12 kilobases downstream (S4 locus marked as the terminator region) of the *APP* gene (**Fig 4A**). We transfected *Tt*ParB-SNAP to visualize the S3 locus and *Tt*ParBm-HaloTag to visualize the S4 locus. As shown in **Fig 4B**, the S3 locus (8×ParSc) and S4 locus (8×ParSm) were visualized simultaneously in the U2OS-8×ParSc-APP-8×ParSm cells. We also test the influence of the ParSite system on gene transcription. The RT-qPCR result reveals that the ParSite system barely affect *APP* gene’s transcriptional activity (**S6 Fig**). As statistical analysis shown in **Fig 4C**, the 2D spatial distance between S3 (promoter region) and S4 (terminator region) ranges from 37 nm to 697 nm with 284 nm on average from 3D projection, which indicates the distance is heterogeneously distributed. Previous research shows that the terminator region will gradually reaches the promoter region, forming a chromatin contact “stripe” feature in the Hi-C map during transcription [34], suggesting the dynamic interaction between the gene’s promoter and terminator. As shown in **Fig 4D**, live cell tracking of the 2D distance from 3D projection between S3 and S4 over 40 s among several cells revealed that promoter and terminator regions of the *APP* gene maintained the spatial distances in each cell. A similar observation was seen when tracking for 8 min (**Fig 4E and S1 Video**).

### A ParSc-ParSm hybrid system for non-repetitive DNA imaging

**Fig 5:**
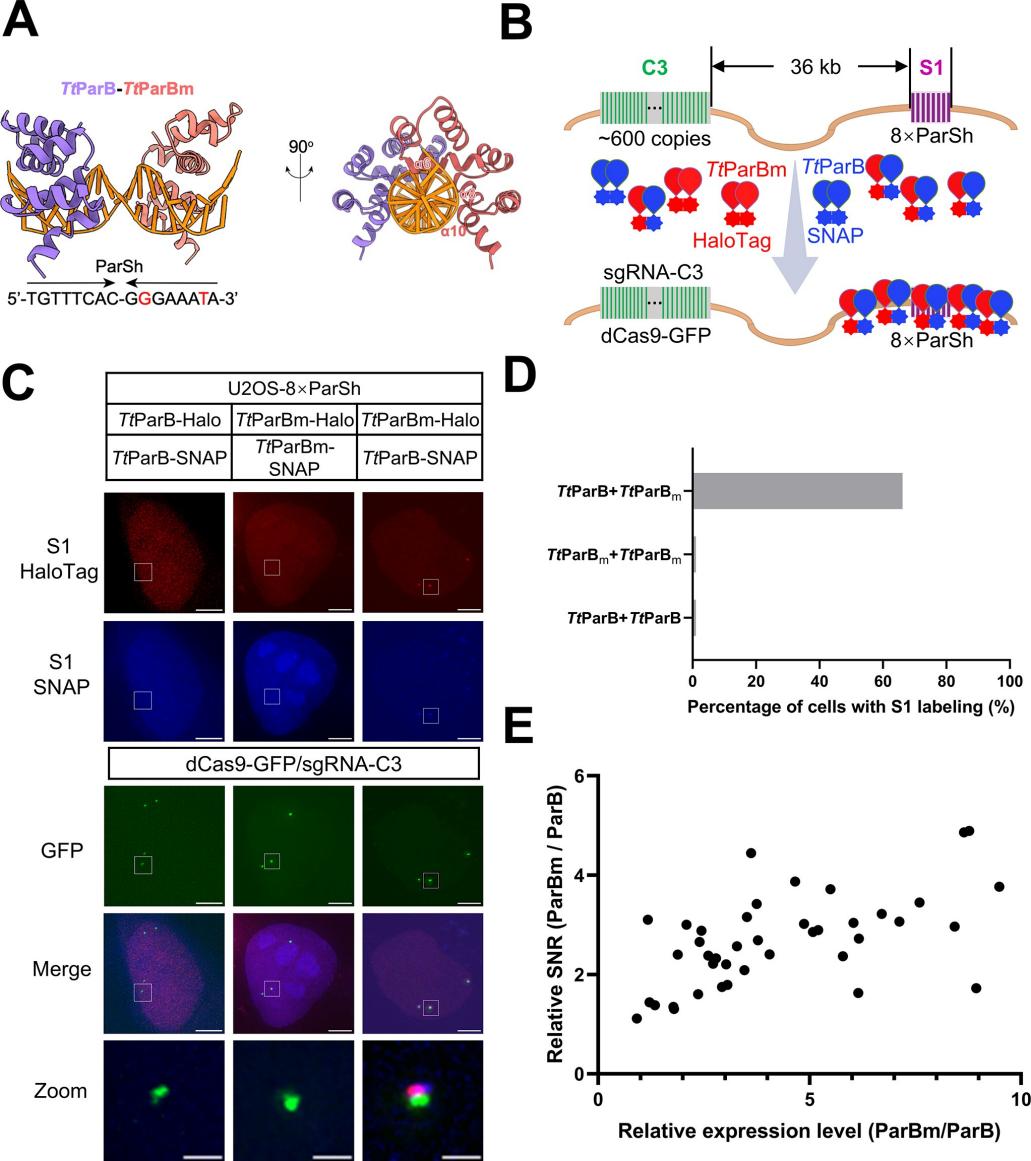
Generation of a hybrid ParB-ParS system for live-cell DNA imaging. (A) Structures of a hybrid ParB-ParS system. The heterodimer of *Tt*ParB (purple) and *Tt*ParBm (red) recognizes a ParS hybrid (ParSh) with a half ParSc and a half ParSm. The organizations of *Tt*ParB and *Tt*ParBm are based on the structure of *Caulobacter vibrioides* ParB (PDB entry 6T1F). The model in the right panel is viewed after a 90° rotation around the vertical axis. The helices that interact with the DNA molecule are labeled as α6, α8, and α10. (B) Diagram of the hybrid ParB-ParS system for DNA imaging. The 8×ParSh is integrated into the S1 locus, which is 36 kb downstream of the C3 repeat (as an endogenous reference locus). The hypothesis is that the heterodimer of *Tt*ParB and *Tt*ParBm will bind ParSh and could be used for DNA imaging. (C) Representative images of U2OS-8×ParSh labeled by *Tt*ParB-*Tt*ParBm heterodimer. *Tt*ParB only group and *Tt*ParBm only group are as controls. Scale bars, 5 μm for images with the whole cells and 1 μm for zoomed images. (D) The percentage of U2OS-8×ParSh cells labeled by *Tt*ParB-*Tt*ParB, *Tt*ParBm-*Tt*ParBm, or *Tt*ParB-*Tt*ParBm dimmers, respectively. *n* = 62 cells. (E) The quantification of ParSh relative labeling efficiency by ParB and ParBm. *n* = 42. One data point is outside the axis limit. The underlying data associated with this figure are available in S5 Data.

Based on the ParB-ParS crystal structures, ParB dimer binds the 16 bp palindromic ParS sequence. *Tt*ParB dimer or *Tt*ParBm dimer binds to 16 base-pair palindromic ParSc or ParSm without cross-reaction. Here, we test whether the hybrid (ParSh) of half-ParSc and half-ParSm could be specifically recognized by *Tt*ParB and *Tt*ParBm heterodimer (**Fig 5A**). We knocked in 8×ParSh at the S1 locus (36 kb downstream of the C3 repeat) in U2OS cells (**S1A and S1B Fig**). We transfected *Tt*ParB and/or *Tt*ParBm to visualize the S1 locus, along with dCas9-GFP/sgRNA-C3 for labeling C3 locus (**Fig 5B**). As shown in **Fig 5C** and **5D**, the S1 locus (8×ParSh in magenta) was not labeled by the *Tt*ParB dimer or *Tt*ParBm dimer but efficiently labeled by the *Tt*ParB-*Tt*ParBm heterodimer. Live tracking the S1 locus containing 8×ParSh, we saw the signal foci of *Tt*ParBm-HaloTag overlapped with *Tt*ParB-SNAP (**S7 Fig**). The signal intensity from ParBm and ParB spreading around ParSh is positively correlated with their relative expression level (**Fig 5E**). We also combine the 8×ParSm and 8×ParSh to achieve dual-color genomic loci labeling (**S8 Fig**). The ParSc-ParSm hybrid data enable us, for the first time, to use ParS label genome DNA without a palindromic sequence. Therefore, 8×ParSc, 8×ParSm, and 8×ParSh could be utilized for triple-color DNA imaging, and we termed this system as tParSite (tricolor ParSite).

### Simultaneously labeling 3 genomic loci by tricolor ParSite

**Fig 6:**
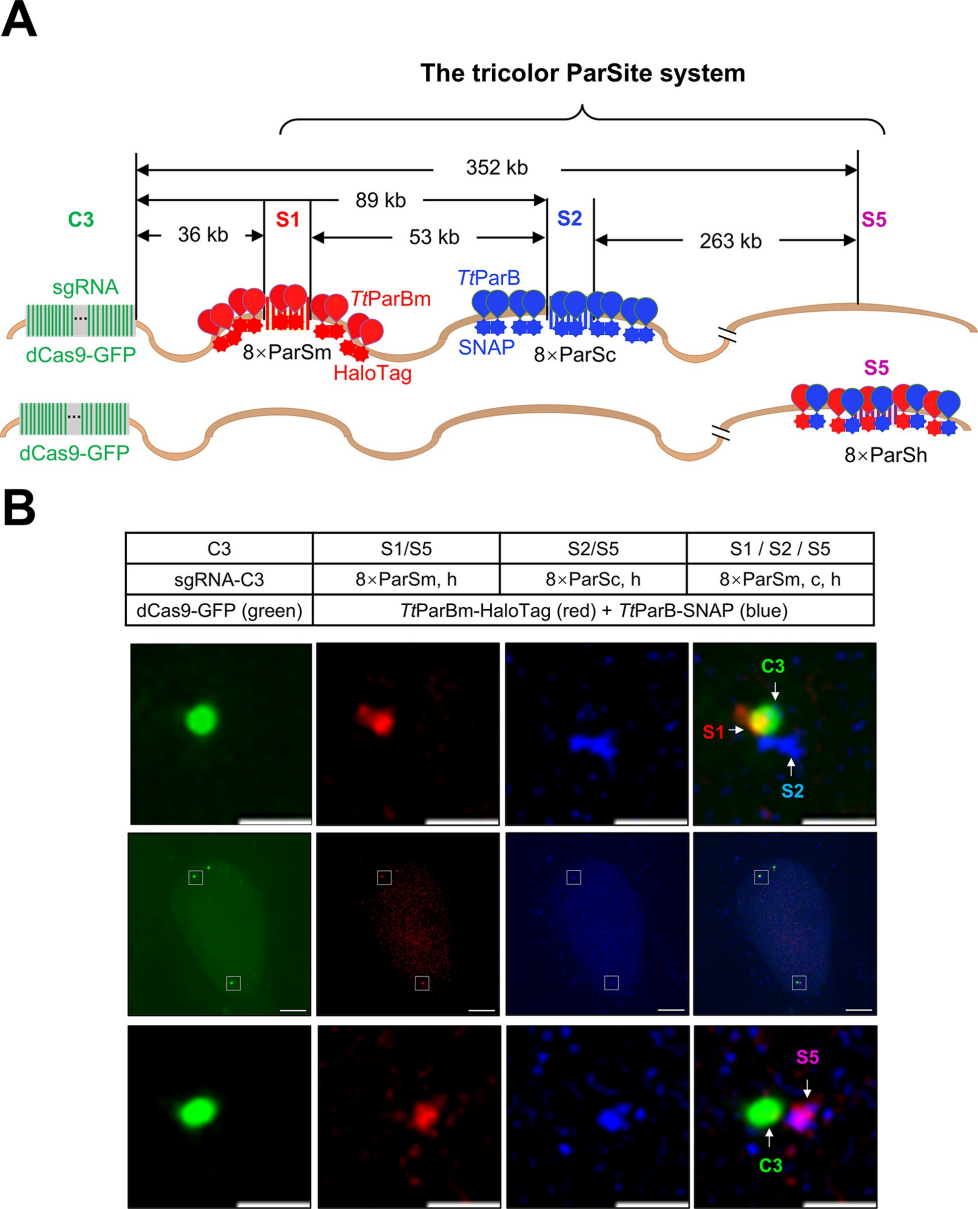
Labeling 3 genomic DNA loci simultaneously by tricolor ParSite. (A) Simplified schematic of the tricolor ParSite system for simultaneous imaging of 3 genomic loci; 8×ParSm, 8×ParSc, and 8×ParSh were integrated into U2OS at the S1, S2, and S5 loci, respectively, downstream of C3 repeat on chromosome 3. The dimeric *Tt*ParBm-HaloTag, dimeric *Tt*ParB-SNAP, and heterodimeric *Tt*ParBm-HaloTag/*Tt*ParB-SNAP were used for labeling S1, S2, and S5, respectively. (B) Representative images of simultaneous imaging of 3 genomic loci in single cell. U2OS-8×ParSm-8×ParSc-8×ParSh cell line was transfected with *Tt*ParB-SNAP and *Tt*ParBm-HaloTag, along with dCas9-GFP and sgRNA-C3 for labeling of C3 repeats as a reference point. The middle panel was the whole cell nucleus. The upper panel shows the labeling of S1 (red) and S2 (blue) loci adjacent to C3 (green). The below panel shows the labeling of S5 (magenta) locus adjacent to C3. Scale bars, 5 μm for images with the whole cells and 1 μm for zoomed images.

To confirm that the tricolor ParSite (tParSite) could simultaneously label 3 target sites, we knocked in 8×ParSm at the S1 locus (36 kb downstream of the C3 locus), 8×ParSc at the S2 locus (89 kb downstream of the C3), and 8×ParSh at the S5 locus (352 kb downstream of the C3 repeat) into U2OS cells. To get the cell lines with knocked in 3 above 8×ParS derived sequences, we established a knock-in method with high integration efficiency resulting in 62% for 8×ParSm, 96.15% for 8×ParSc, and 100% for 8×ParSh (**S1 Fig**). We transfected *Tt*ParBm-HaloTag to visualize the S1 locus, *Tt*ParB-SNAP to visualize the S2 locus, *Tt*ParBm-HaloTag/*Tt*ParB-SNAP together to visualize the S5 locus, along with dCas9-GFP/sgRNA-C3 for labeling C3 repeat (**Fig 6A**). As shown in the **top row of Fig 6B**, C3 (green) and S1 (red) are partially overlapped, and both of them are proximal to S2 (blue). As shown in the **bottom row of Fig 6B**, C3 is proximal to S5 (purple was generated from red and blue overlapping). We also live-tracked the S1, S2, and S5 in the single cell (**S2 and S3 Videos**). These data suggested we generated a U2OS cell line with one allele containing 8×ParSm at S1 and 8×ParSc at S2 and another allele containing 8×ParSh at S5, which can achieve 3 color DNA imaging with ParSc, ParSm, ParSh, *Tt*ParB, and *Tt*ParBm. The labeling efficiency is 33.3% for tParSite (**S4 Fig**). It is easier to separate the 3 DNA loci of ParSc, ParSm, and ParSh if they located on different chromosomes than on one chromosome. Live cell tracking is needed if these 3 loci integrated into same chromosome, and the distance between a pair of them is critical for later spot analysis.

## Discussion

Genome organization in space and time is critical for transcriptional regulation, cell growth, and differentiation. There are extensive studies on genome organization in 3D in fixed cells by either sequencing such as Hi-C, or imaging such as DNA FISH [35]. However, the studies on genome organization in 4D (time is the fourth dimension) are lacking behind, mainly because of the challenge of imaging non-repetitive genomic loci in living cells. We previously developed mParSpot [30], a dual-color DNA imaging approach based on the ParB-ParS and Noc-NBS systems. The mParSpot allows to track pairwise genomic loci and explore genomic interactions. Here, we created ParSite, a multicolor DNA imaging approach by a two-step process: (1) rational design of the interface amino acids between *Tt*ParB and ParSc resulting in *Tt*ParBm and ParSm; (2) Generation of the ParSh with half-ParSc and half-ParSm allowing for non-repetitive genomic DNA imaging. Combining ParB-ParSc and ParBm-ParSm, we can realize dual color DNA imaging. Although we are unclear of the impact of ParSite on genome topology, ParSite hardly down-regulate gene transcription in our cell lines. Based on ParSc, ParSm, ParSh, ParB, and ParBm, we can achieve triple-color DNA imaging. Importantly, the tricolor ParSite will allow us to track the contour of multiple loci on chromosomes in living cells. This will be important to provide information on DNA loop dynamics at a single-cell level over time.

ParB typically spreads a few kilobases around ParS from Chip-seq in bacteria [36]. The number of ParB proteins around the ParS site for the DNA labeling in mammalian cells is worthwhile considering. To estimate the amount of ParB proteins around the integrated ParS site, we compared the intensity of TetR on 120×TetO with ParB around 8×ParS. It was estimated an average of 52 ParB or 30 ParBm proteins around the ParSc or ParSm site, respectively (**S9 Fig**). The spot size of ParB around the ParS site was measured to be less than 300 nm under the wide-field microscope we used. The spot size and shape could be more precise if we could use the super-resolution microscope for live cell DNA imaging by the ParSite system in future studies. The SNR is negatively correlated with the expression level of ParB, which indicates that background reduction could also enhance the sensitivity of DNA imaging by the ParSite system.

ParB and Noc are structurally similar, and the DNA-binding specificity of ParB to ParS can switch to NBS by mutations of 4 conserved amino acids [31]. However, it is unclear whether *Tt*ParB containing the above 4 mutations (*Tt*ParBm) can switch its binding specificity from ParSc to NBSc (ParSm), which is 4 nucleotides different in between, for DNA imaging in living cells. We found these mutations could successfully switch the binding specificity of *Tt*ParB for DNA imaging, although the SNRs decreased to some degree. To our surprise, the ParSh of half-ParSc and half-ParSm switches its binding specificity to the heterodimer *Tt*ParB and *Tt*ParBm, which led us to develop tricolor ParSite. These results, for the first time, demonstrated heterodimer ParB was able to recognize non-palindromic ParS hybrid and spread around the neighboring DNA. The detailed mechanism for the recognition and spreading of ParBs around the ParS site is unknown. We hypothesize that it might be the ParB-ParBm heterodimer recognition of the ParSh site and subsequently spreading around the same spot. Previously, we found the Noc-NBS system could be repurposed for DNA imaging [30]. To further expand the labeling colors for DNA loci, we developed the ParSite system to achieve triple-color DNA imaging. By adding one more color, we can not only measure the distance of 2 genomic loci over time but also potentially track the contour of 3 genomic locations in space and time. To better locate the 3 genomic spots labeled by tParSite, time-lapse tracking is useful for resolving the colocalization concern if they are close in genomic distance or spatial distance. To be concluded, the ParSite system is a simple and compact tool for DNA imaging in living cells with minimal disturbance in RNA transcription and cell growth.

It has been proposed that loop interactions among enhancers, promoters and terminators regulate the transcription of genes [34,37–41]. However, it is still not fully understood how the spatial distance and duration of promoter and enhancer (P-E) or promoter and terminator (P-T) interactions contribute to transcriptional activity in living cells. Here, we found that the spatial distance of *APP*’s promoter and terminator was maintained during the duration of 40 s, suggesting that the *APP*’s promoter and terminator loops are relatively stable in a short period. It will be intriguing to investigate whether the spatial distances and stability of promoter and terminator loops contribute to the transcriptional activity of the *APP* gene. Using tricolor ParSite (tParSite), we should be able to better define P-T loops with additional labeling of a locus in between the promoter and terminator. By combining with Cas13-based RNA imaging [42], we may investigate how the P-T loops contribute to transcriptional activity or bursts. In sum, the ParSite system is a useful addition to the DNA imaging toolbox and is complementary to the FROS system and CRISPR-based DNA imaging methods. With this strategy, introducing one more ParS independent of ParSc and ParSm could generate a color palette of ParB-ParS-based DNA imaging in living cells.

## Methods

### Plasmid construction

For single color genomic locus labeling, the donor plasmids used for the integration of 8×ParSc, 8×ParSm, and 8×ParSh consist of 4 regions: left homology arm, 8×ParSc, 8×ParSm, or 8×ParSh, puromycin resistant gene with CMV promoter and SV40 PolyA signal and right homology arm. For dual-color genomic loci tracking, the donor plasmids are similar as above, except that the hygromycin-resistant gene with EFS promoter was added as a second selection marker. For triple color DNA labeling, the bleomycin-resistant gene was used as a third selection marker. For gene promoter and terminator labeling, the donor plasmids have 4 regions: left homology arm, 8×ParSc or 8×ParSm, right homology arm, and inverse-oriented CMV promoters. All these donor plasmids were constructed from pDONOR3.1 and ligated with a seamless assembly enzyme mix. For *Tt*ParB or *Tt*ParBm expression vectors, pHAGE-EFS-MCP-HaloTag or pHAGE-EFS-N22P-SNAP was used for subcloning. The 53BP1 DNA fragments were codon-optimized and synthesized by Beijing Tsingke Biotech Co., Ltd. We synthesized a 30-mer TetO array from GenScript. The 120-mer TetO array was constructed with 4 copies of the 30-mer TetO array in a DONOR plasmid. Later, the CMV promoter and enhancer, Puromycin resistant gene, and SV40 PolyA fragments were integrated downstream of the 120-mer TetO array using seamless assembly. Finally, the left homology arm and right homology arm for *DHFR* or *WEE1* are inserted into the 120-mer TetO plasmid, respectively. The core sequences of 8×ParS, 8×ParSm, 8×ParSh, and 120-mer TetO are listed in S1 Table. The sgRNA sequences are listed in S2 Table.

### Generation of U2OS cell lines with 8×ParS integration

U2OS cells were cultured on 10 cm dishes at 37°C in DMEM with high glucose (Life Technologies) supplemented with 10% (vol/vol) FBS. To generate single 120-mer TetO and 8×ParS-integrated cell lines, U2OS cells were co-transfected with 1 μg of pHAGE-Cas9-P2A-sfGFP, 600 ng of pLB-sgRNA1 and 500 ng of pDONOR-8×ParSc, pDONOR-8×ParSm, pDONOR-8×ParSh, or pDONOR-120×TetO using Lipofectamine 2000 (Life Technologies) for 6 h and then replaced with fresh culture media. The transfected cells were cultured for an additional 48 h before flow cytometry to sort BFP and GFP double-positive cells by FACSAria III. The collected cells were plated on 48-well plates and cultured for an additional 24 h. Puromycin was added to enrich the cells with 8×ParSc, 8×ParSm, 8×ParSh, or 120×TetO integration for 7 days. Single cells were then sorted into 96-well plates and cultured for an additional 2 to 3 weeks. The single-cell clones were expanded and subjected to genotyping. To generate dual 8×ParS-integrated cells, the cell line was subjected for double selections with puromycin and hygromycin. To generate triple 8×ParS-integrated cells, the cells were subjected for triple selections with puromycin, followed by hygromycin and zeocin sequentially. Single-cell clones were identified with primers in S3 Table. We used 2 pairs of primers to identify whether the homozygous or heterozygous integration of 8×ParS into the cell lines.

### Transfection for DNA imaging by the ParSite system

For 8×ParSc or 8×ParSm labeling test, 600 ng of SgRNA-C3 repeat, 300 ng of dCas9-GFP, and 500 ng of *Tt*ParB-HaloTag or *Tt*ParBm-HaloTag are transfected together into U2OS cells using Lipofectamine 2000 (Life Technologies). For 8×ParSh-mediated DNA imaging, 8×ParSc and 8×ParSm-mediated dual color DNA imaging or 8×ParSc, 8×ParSm, and 8×ParSh-mediated triple color DNA imaging, 600 ng of sgRNA-C3 repeat, 300 ng of dCas9-GFP and 600 ng of *Tt*ParB-SNAP and/or 500 ng of *Tt*ParBm-HaloTag are transfected together into cells.

### DNA double-strand breaks detection in live cells by GFP-53BP1

We chose 53BP1 as a marker for DNA DSBs in live cells. The locus of downstream C3 repeat and/or upstream S2 were cleavage sites. The day before transfection, S1-ParSm-S2-ParSc-integrated cell lines were cultured into 35 mm glass bottom dish from 10 cm culture dish, and 400 ng of sgRNA-C3 repeat, 1.5 μg of SpCas9-BFP-U6-sgRNA1-U6-sgRNA2, 400 ng of *Tt*ParBm-HaloTag, 1 μg of *Tt*ParB-SNAP, and 300 ng of GFP-53BP1 were transfected together into S1-ParSm-S2-ParSc integrated cells using EZ Trans Transfection Reagent or Lipofectamine 2000 for 6 h, and then replaced with fresh culture media for additional 18 h. HaloTag-JF549 and SNAP-Cell 647-SiR were added into the dish 8 h before imaging. For the control group, 400 ng of sgRNA-C3 repeat, 331 ng of SpCas9-BFP, 400 ng of *Tt*ParBm-HaloTag, 1 μg of *Tt*ParB-SNAP, and 300 ng of GFP-53BP1 were transfected into S1-ParSm-S2-ParSc cells.

### Tracking the promoter and terminator interactions of the *APP* gene by the ParSite system

For promoter and terminator labeling, 600 ng of *Tt*ParB-SNAP and 500 ng of *Tt*ParBm-HaloTag were transfected together into ParS-integrated cells using EZ Trans Transfection Reagent for 8 h and replaced with fresh culture media then for 24 h before imaging.

### RT-qPCR

The U2OS-8×ParSc-APP-8×ParSm cells were cultured in a 6-cm dish, and 1.3 μg of *Tt*ParB-HaloTag or control plasmids were transfected into cells. One day after transfection, cells were collected to sort transfected cells by Arial III. Total RNA was extracted using the RNAprep Pure Micro Kit (Tiangen Biotech Co., Ltd.). We utilized ABScript III RT Master Mix for qPCR with gDNA Remover and 2X Universal SYBR Green Fast qPCR Mix (ABclonal) to do RT-qPCR reaction. The primer sequences are listed in S3 Table.

### Triple color DNA imaging in live cells by tParSite system

Approximately 16 h after transfection, HaloTag-JF549 and SNAP-Cell 647-SiR were added to the dish. The image is acquired 24 h after transfection. To verify and separate the triple spots labeled by tParSite system (ParB/ParSc, ParBm/ParSm, ParB-ParBm/ParSh), we recommend testing the comovement of ParB and ParBm on each locus by live tracking. For ParSc and ParSm, the fluorescence signals of ParB and ParBm would separate most of the time or at a certain time during tracking. For ParSh, the fluorescence signals of ParB and ParBm would be most likely overlapped all the time during tracking.

### Fluorescence microscopy

The live-cell DNA imaging was carried out on a DeltaVision Ultra imaging system equipped with a 100× PlanApo oil objective lens (NA 1.4). The cells were cultured on No. 1.0 glass bottom dishes (MatTek). BFP was excited with an excitation filter at 397/31 nm, and its emission was collected using an emission filter at 438/36 nm. sfGFP was excited at 478/28 nm and collected using the filter at 512/23 nm. HaloTag-JF549 was excited at 548/34 nm, and its emission was collected using the filter at 592/38 nm. SNAP-Cell 647-SiR was excited with an excitation filter at 633/27 nm, and its emission was collected using an emission filter at 677/46 nm. The fluorescence imaging data were acquired by DeltaVision Elite imaging software. The images were captured in z-stacks with an exposure time of 50 ms under 50% laser power for HaloTag, GFP, BFP, or SNAP, respectively. The step size in z-stacks was 200 nm. For the representative images, the raw data were deconvoluted and projected by softWoRx software.

### Statistical analysis

The bar graph, dot plot, and broken line chart are produced by GraphPad Prism software. Experiments with representative data are conducted at least 3 times unless otherwise stated. Images are randomly captured in different fields of interest and processed one by one, excluding non-complete or worse pictures. Data are processed with Excel. The labeling efficiency is estimated from all transfected cells. The SNR was calculated with the formula: SNR = (I_S_-I_B_)/(I_N_-I_B_). I_S_ is the intensity of the labeled loci; I_N_ is the intensity of the nucleoplasm; and I_B_ is the background fluorescence intensity from a dark region in the same image.

The zoom-in images are processed in ImageJ by adjusting the minimum and maximum fluorescence intensity range. The background fluorescence intensity of proteins is calculated with I_N_-I_B_ (the I_N_ and I_B_ are mentioned above) and measured by ImageJ. The 2D distances are calculated with the formula $d=\sqrt[{2}]{{\left(X2-X1\right)}^{2}+{\left(Y2-Y1\right)}^{2}}$ in Excel. The position of the spot is measured by ImageJ. The spot area is marked with an irregular shape around its contour and measured by ImageJ. The 3D distances are calculated by Imaris with its spot module.

The percentage of cells grouping in Fig 3B–3D is calculated within cells containing 53BP1 foci, and the foci are close or overlap with C3 or S2 spots.

Relative SNR in Fig 5E is calculated with the formula: relative SNR = (I_S_-I_B_)_ParBm_/(I_S_-I_B_)_ParB_. The relative expression level of ParBm versus ParB is calculated with the formula: Relative expression level_ParBm/ParB_ = (I_N_-I_B_)_ParBm_/(I_N_-I_B_)_ParB_. I_S_ is the signal fluorescence intensity for ParB or ParBm at the ParSh site. I_N_ is the nuclear intensity of ParBm or ParB expressed cells. I_B_ is the fluorescence intensity of the dark region in the same image.

The mean intensity of the signal spot in S9 Fig is calculated with the formula: mean intensity = I_S_-I_B_. I_S_ is the fluorescence intensity of signal foci, and I_B_ is the fluorescence intensity of the dark region in the same image.

## Supporting information

S1 Table The core sequences of 8×ParSc, 8×ParSm, 8×ParSh, and 120×TetO. (DOCX)

S2 Table The sequences of SgRNA. (DOCX)

S3 Table The primers used in this study. (DOCX)

S1 Fig Integration of 8×ParS adjacent to C3 repeat in the U2OS cells. (A) The schematic of sequential integration of 8×ParSs into different genomic target sites. The 8×ParS-1was firstly integrated into a target site along with the expression cassette of selection marker puromycin. The 8×ParS-2 was integrated into another target site along with the expression cassette of selection marker hygromycin. The 8×ParS-3 was integrated into a third target site along with the expression cassette of selection marker zeocin. (B) Clonal selection by FACS. Donor plasmids containing 8×ParS-1 and puromycin resistant gene along with Cas9/sgRNA were transfected into U2OS cells. Puromycin was used to kill these cells without transfected and FACS was applied for the single-cell selection. The second donor plasmid contains 8×ParS-2 and hygromycin resistant gene and hygromycin was used to select 8×ParS-2 integrated cells. The third donor plasmid contains 8×ParS-3 and zeocin resistant gene. Zeocin was used to select 8×ParS-3 integrated cells. Through these steps to improve integration efficiency. (C) Integration efficiency of 8×ParS into U2OS cells. The integration efficiency was estimated by counting the positive clones with 8×ParSm, 8×ParSc, or 8×ParSh from passaged single-cell clone. The underlying data associated with this figure are available in S6 Data.
(TIF)

S2 Fig The correlation of SNR and ParB expression. (A) The quantification of SNR from ParB/ParS system by the expression level of ParB protein. *n* = 50. (B) The quantification of SNR from ParBm/ParSm by the expression level of ParBm protein. *n* = 46. The underlying data associated with this figure are available in S7 Data.
(TIF)

S3 Fig The 3D distance and area of C3, S1, and S2 loci. (A) The representative zoom-in images of C3 (green), S1 (brown), and S2 (red) spots. The left is the original processed image and the right is the spot-module mimic image. Scale bars, 0.2 μm. (B) The distribution of 3D distance with C3-S1, S1-S2, and C3-S2. Red lines indicate the mean value. *n* = 42. (C) The area measurement of C3, S1, and S2 spots. *n* = 37. The red line indicates the mean area of each group. The underlying data associated with this figure are available in S8 Data.
(TIF)

S4 Fig The labeling efficiency of double- and triple-color DNA labeling. (A) The diagram of double and triple color DNA labeling. (B) Labeling efficiency of double- and triple-color DNA imaging. *n* = 42 cells for double-label, *n* = 42 cells for triple-label. The underlying data associated with this figure are available in S9 Data.
(TIF)

S5 Fig The representative images of dParSite cells when no cleavage is induced. SgRNA-C3, SpCas9-BFP, GFP-53BP1, ParBm-HaloTag, and ParB-SNAP plasmids are transfected together into cells. Arrowheads indicate the positions of C3, S1, and S2 loci. Scale bars, 5 μm.
(PDF)

S6 Fig Fold change of *APP* mRNA level in the control group and ParS/ParB group. We transfect *Tt*ParB plasmid or control plasmid into U2OS-8×ParSc-APP-8×ParSm cells. Total RNA is extracted to complete RT-qPCR testing the expression level of *APP* gene. The underlying data associated with this figure are available in S10 Data.
(TIF)

S7 Fig Live cell tracking of the S1 locus labeled by the ParB-ParS hybrid system. (A) The diagram of labeling the S1 locus by the ParB-ParS hybrid system; 8×ParSh was integrated into the S1 locus and labeled by the heterodimer of *Tt*ParB and *Tt*ParBm. (B) Time-lapse of the S1 locus labeled by the ParB-ParS hybrid system. Both *Tt*ParBm-HaloTag and *Tt*ParB-SNAP labeled the S1 locus in the ParB-ParS hybrid system. The S1 locus was tracked over 56 s. Scale bars, 0.5 μm. (C) Plots of the fluorescence intensity of the S1 locus indicated by the dashed lines in B. Red line represents the fluorescence intensity of *Tt*ParBm-HaloTag. Blue line represents the fluorescence intensity of *Tt*ParB-SNAP. The length of each dashed line is 0.7 μm.
(TIF)

S8 Fig Simultaneous imaging of 2 DNA loci by 8×ParSm and 8×ParSh. (A) The schematic of labeling S1 and S5 loci by ParSm or ParSh; 8×ParSm and 8×ParSh were integrated into U2OS at the S1 and S5 loci respectively, downstream of C3 repeat on chromosome 3. The dimeric *Tt*ParBm-HaloTag and heterodimeric *Tt*ParBm-HaloTag/*Tt*ParB-SNAP were used for labeling S1 or S5, respectively. (B) Representative images of simultaneous imaging of the S1 and S5 loci. *Tt*ParB-SNAP and *Tt*ParBm-HaloTag along with dCas9-GFP and sgRNA-C3 for labeling of C3 repeats are transfected into cells to label S1, S5, and C3 repeat loci. The bottom panel shows the simultaneous labeling of C3 (green), S1 (red), and S5 (magenta). Scale bars, 5 μm for images with the whole cells and 1 μm for zoomed images.
(TIF)

S9 Fig The intensity comparison of TetO/TetR DNA labeling system and ParB/ParS system. (A) The labeling strategy for DHFR and WEE1 genes by 120-mer TetO array. (B) Representative images of DHFR and WEE1 genes labeling with TetO/TetR system. Scale bars, 5 μm. (C) The SNR of 120-mer TetO/TetR system. *n* = 29 for DHFR group, and *n* = 40 for WEE1 group. Red lines indicate mean value. (D) The mean intensity of signal foci for 120-mer TetO_DHFR_/TetR, 120-mer TetO_WEE1_/TetR, 8-mer ParSc/ParB, and 8-mer ParSm/ParBm systems. *n* = 32, 39, 48, 46 for each group from left to right. Red line indicates mean value for each group. The underlying data associated with this figure are available in S11 Data.
(TIF)

S1 Video Tracking the dynamics of promoter and terminator region of *APP* gene by ParSite. The video is shown in a total time of 8 min. The interval between each picture is 1 min. Images were cropped to 50 × 50 pixels. Scale bar, 1 μm. The playback rate is 5 frames per second. Green: S3 locus (promoter region) and Red: S4 locus (terminator region).
(MP4)

S2 Video Time-lapse tracking of S1 and S2 loci in tParSite cells. The video scan time is 66 s. The scan sequence is first Channel then Z to minimize time delay effect. Images were cropped to 50 × 50 pixels. Scale bar, 1 μm. The playback rate is 10 frames per second. Blue: S2 locus, Green: C3 locus, Red: S1 locus.
(MP4)

S3 Video Time-lapse tracking of S5 loci in tParSite cells. The video scan time is 66 s. The scan sequence is first Channel then Z to minimize time delay effect. Images were cropped to 50 × 50 pixels. Scale bar, 1 μm. The playback rate is 10 frames per second. Green: C3 locus, magenta (blue + red): S5 locus.
(MP4)

S1 Data Numerical data used for the generation of the graphs presented in Fig 1. (XLSX)

S2 Data Numerical data used for the generation of the graphs presented in Fig 2. (XLSX)

S3 Data Numerical data used for the generation of the graphs presented in Fig 3. (XLSX)

S4 Data Numerical data used for the generation of the graphs presented in Fig 4. (XLSX)

S5 Data Numerical data used for the generation of the graphs presented in Fig 5. (XLSX)

S6 Data Numerical data used for the generation of the graphs presented in S1 Fig. (XLSX)

S7 Data Numerical data used for the generation of the graphs presented in S2 Fig. (XLSX)

S8 Data Numerical data used for the generation of the graphs presented in S3 Fig. (XLSX)

S9 Data Numerical data used for the generation of the graphs presented in S4 Fig. (XLSX)

S10 Data Numerical data used for the generation of the graphs presented in S6 Fig. (XLSX)

S11 Data Numerical data used for the generation of the graphs presented in S9 Fig. (XLSX)

## Abbreviations

DSB: double-strand break
FROS: fluorescence repressor operator system
LLPS: liquid–liquid phase separation
SNR: signal-to-noise ratio
TAD: topologically associating domain
