# Supplementary material for: ParSite is a multicolor DNA labeling system that allows for simultaneous imaging of triple genomic loci in living cells
Source: PLoS Biol. 2025 Jan 24;23(1):e3003009. doi: 10.1371/journal.pbio.3003009 (PMC11798528; doi:10.1371/journal.pbio.3003009)
Supplement: S1 Table — (DOCX) [file pbio.3003009.s001.docx]

|  | Core sequence | Description |
| --- | --- | --- |
| 8×ParSc | TGTTTCACGTGAAACA | Core sequence for imaging with 32nt spacer sequence |
| 8×ParSm | TATTTCCCGGGAAATA | Core sequence for imaging with 32nt spacer sequence |
| 8×ParSh | TGTTTCACGGGAAATA | Core sequence for imaging with 32nt spacer sequence |
| 120×TetO | TCCCTATCAGTGATAGAGA | Core sequence for imaging with 10nt spacer sequence |

S1 Table. The core sequences of 8×ParSc, 8×ParSm, 8×ParSh and 120×TetO.
