# Supplementary material for: ParSite is a multicolor DNA labeling system that allows for simultaneous imaging of triple genomic loci in living cells
Source: PLoS Biol. 2025 Jan 24;23(1):e3003009. doi: 10.1371/journal.pbio.3003009 (PMC11798528; doi:10.1371/journal.pbio.3003009)
Supplement: S2 Table — (DOCX) [file pbio.3003009.s002.docx]

| SgRNA | Spacer sequence | Description |
| --- | --- | --- |
| S1 | GCTCCTCGTCGACATTCCCG | The knock-in site for S1 |
| S2 | AGTCACTTCCGCTCACATGG | The knock-in site for S2 |
| S3 | CTGTATTGCCTTTAATCATC | The knock-in site for S3 |
| S4 | GTCAACTTGCGTGCAGTCCC | The knock-in site for S4 |
| S5 | AGAACGGTGAACGCCAGGTA | The knock-in site for S5 |
| Sg-5 | AGTAGGGACATCTTCACATG | Cleavage downstream from C3 repeat |
| Sg-10 | AGCACCAAAATGCAGGACTC | Cleavage upstream from S2 |
| Sg-DHFR | TTCTGTAGCCTATATCGGGA | The knock-in site for 120×TetO_DHFR_ |
| Sg-WEE1 | TGTAAATCTTAAGTGAAGCC | The knock-in site for 120×TetO_WEE1_ |

S2 Table. The sequences of SgRNA.
