# Supplementary material for: ParSite is a multicolor DNA labeling system that allows for simultaneous imaging of triple genomic loci in living cells
Source: PLoS Biol. 2025 Jan 24;23(1):e3003009. doi: 10.1371/journal.pbio.3003009 (PMC11798528; doi:10.1371/journal.pbio.3003009)
Supplement: S3 Table — (DOCX) [file pbio.3003009.s003.docx]

|  | Primer sequence (5’-3’) | Description |
| --- | --- | --- |
| S1-F | ACTAATGGACTTCCGGTCGTT | Screening for S1 site integration |
| S1-R | GAGGCGATTCCAATGCAGAC | Screening for S1 site integration |
| S1-middle-R | CCCCGTAATTGATTACTATTAATAACTAGT | Screening for S1 site integration |
| S2-F | GCAGGGGATTGCCAAGTAGT | Screening for S2 site integration |
| S2-R | CTGGAATGTTCACTATGCCAC | Screening for S2 site integration |
| S2-middle-R | cacggcgactactgcactta | Screening for S2 site integration |
| S3-F | TTGCTGCACCAACTCCTAGTG | Screening for S3 site integration |
| S3-R | TATCTGAGAAAAGAACCTGGC | Screening for S3 site integration |
| S4-F | GCAGGGCAAGGACAACTAATG | Screening for S4 site integration |
| S4-R | AAGAGAGACGAGTTCCTGTAG | Screening for S4 site integration |
| S4-5-middle-F | cgtctcgactcgcggccgc | Screening for S4 and S5 site integration |
| S4-5-middle-R | gcggccgcgagtcgagacg | Screening for S4 and S5 site integration |
| S5-F | TGGGCTGACTCCCTTTAAGC | Screening for S5 site integration |
| S5-R | CAGCTCGAGGTTTCTCAGGG | Screening for S5 site integration |
| DHFR-test-F | GTTGCCATCCTTCAACGCAATAAG | Screening for 120-mer TetO integration |
| DHFR-test-R | AGTAGATCTGCAGGTCCTGATACTG | Screening for 120-mer TetO integration |
| WEE1-test-F | TGCGGTTGGGTAGGAGGTAT | Screening for 120-mer TetO integration |
| WEE1-test-R | TCCAGGCCTGGAAACCTATTTCATG | Screening for 120-mer TetO integration |
| TetO-middle-F | cgtcagatccgctagaGCTAGC | Screening for 120-mer TetO integration |
| GAPDH-F | CAATGACCCCTTCATTGACC | Primer for RT-qPCR |
| GAPDH-R | TTGATTTTGGAGGGATCTCG | Primer for RT-qPCR |
| APP-qp-F | ccttctcgttcctgacaagtgc | Primer for RT-qPCR |
| APP-qp-R | ggcagcaacatgccgtagtcat | Primer for RT-qPCR |

S3 Table. The primers used in this study.
