# Supplementary figures and images for: ParSite is a multicolor DNA labeling system that allows for simultaneous imaging of triple genomic loci in living cells
Source: PLoS Biol. 2025 Jan 24;23(1):e3003009. doi: 10.1371/journal.pbio.3003009 (PMC11798528; doi:10.1371/journal.pbio.3003009)

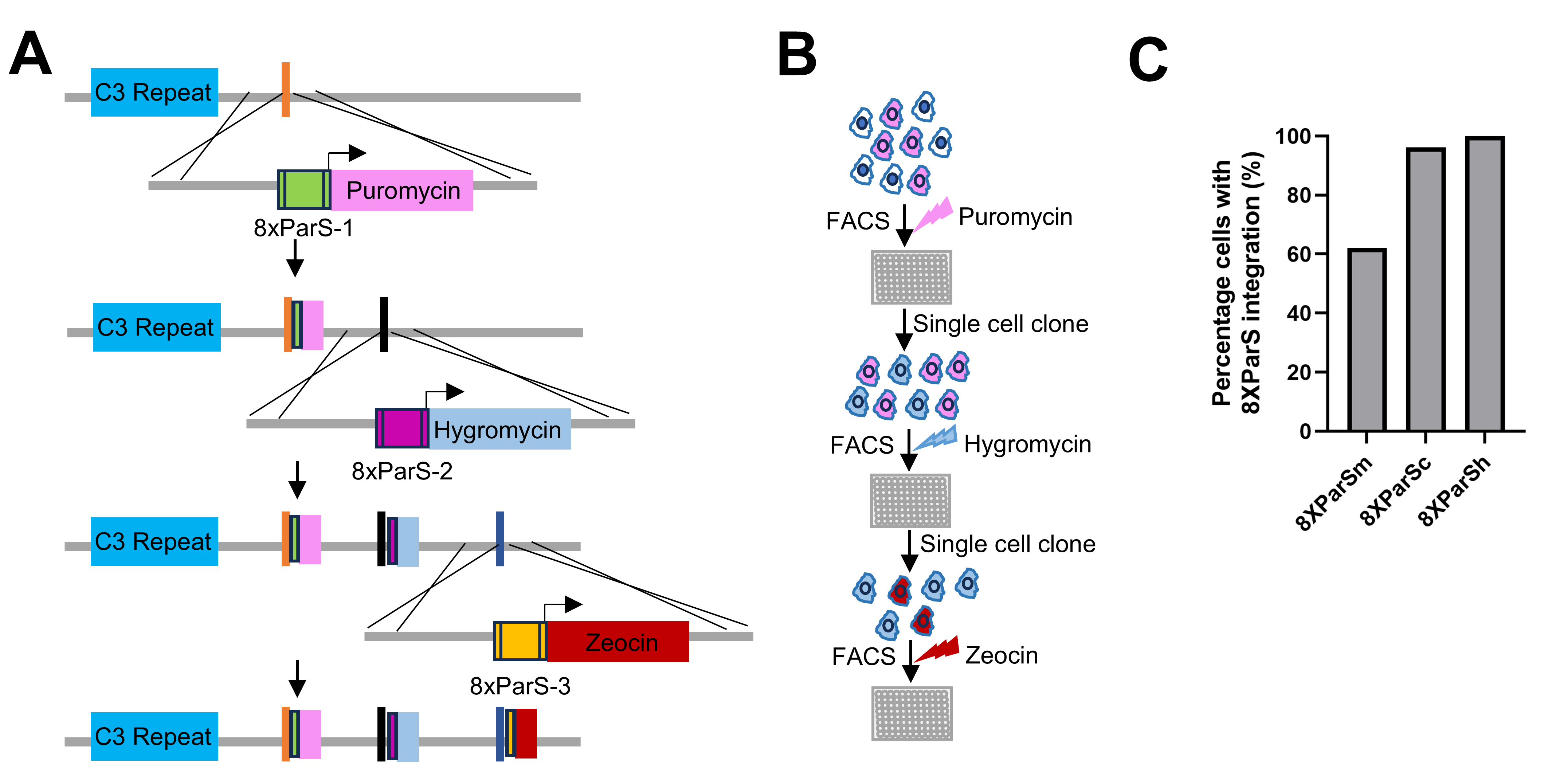

Supplement: S1 Fig — (A) The schematic of sequential integration of 8×ParSs into different genomic target sites. The 8×ParS-1was firstly integrated into a target site along with the expression cassette of selection marker puromycin. The 8×ParS-2 was integrated into another target site along with the expression cassette of selection marker hygromycin. The 8×ParS-3 was integrated into a third target site along with the expression cassette of selection marker zeocin. (B) Clonal selection by FACS. Donor plasmids containing 8×ParS-1 and puromycin resistant gene along with Cas9/sgRNA were transfected into U2OS cells. Puromycin was used to kill these cells without transfected and FACS was applied for the single-cell selection. The second donor plasmid contains 8×ParS-2 and hygromycin resistant gene and hygromycin was used to select 8×ParS-2 integrated cells. The third donor plasmid contains 8×ParS-3 and zeocin resistant gene. Zeocin was used to select 8×ParS-3 integrated cells. Through these steps to improve integration efficiency. (C) Integration efficiency of 8×ParS into U2OS cells. The integration efficiency was estimated by counting the positive clones with 8×ParSm, 8×ParSc, or 8×ParSh from passaged single-cell clone. The underlying data associated with this figure are available in S6 Data. (TIF) [file pbio.3003009.s004.TIF]

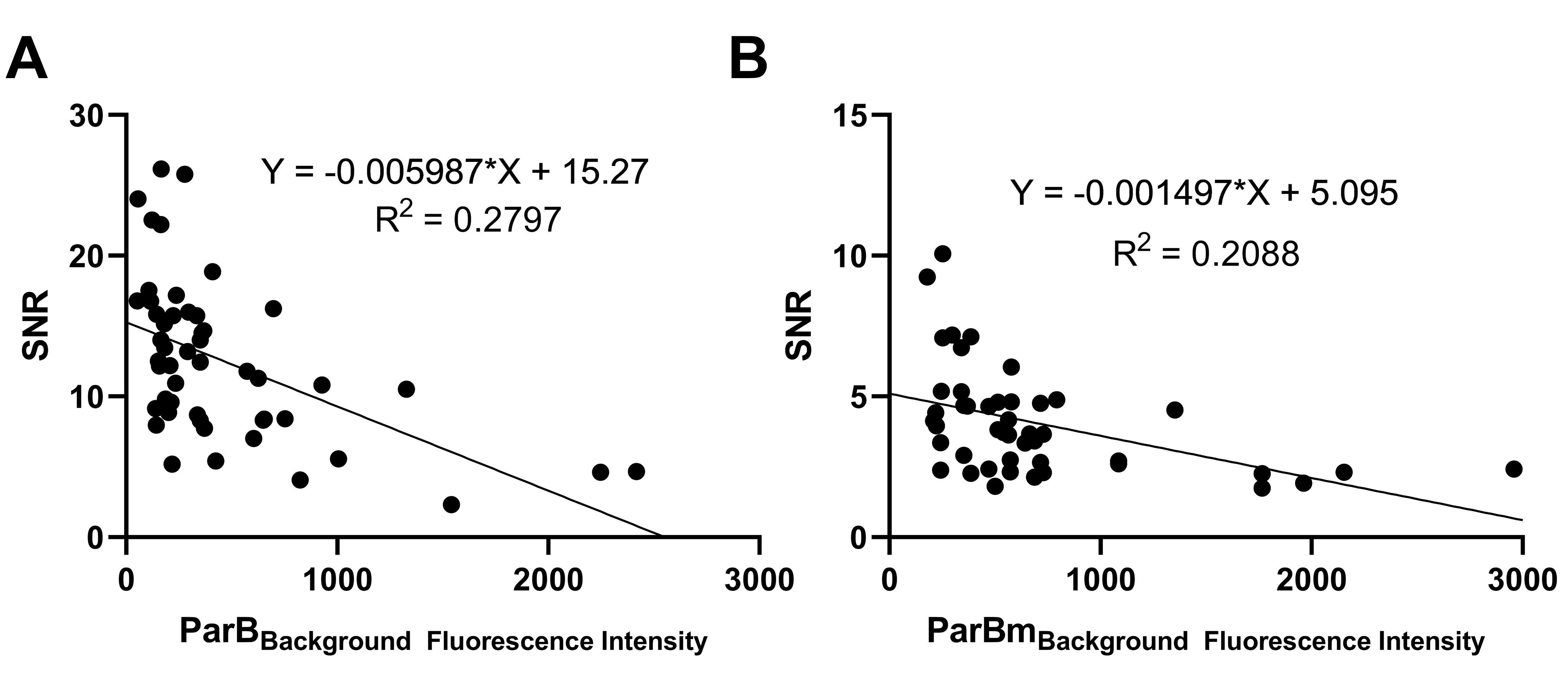

Supplement: S2 Fig — (A) The quantification of SNR from ParB/ParS system by the expression level of ParB protein. n = 50. (B) The quantification of SNR from ParBm/ParSm by the expression level of ParBm protein. n = 46. The underlying data associated with this figure are available in S7 Data. (TIF) [file pbio.3003009.s005.TIF]

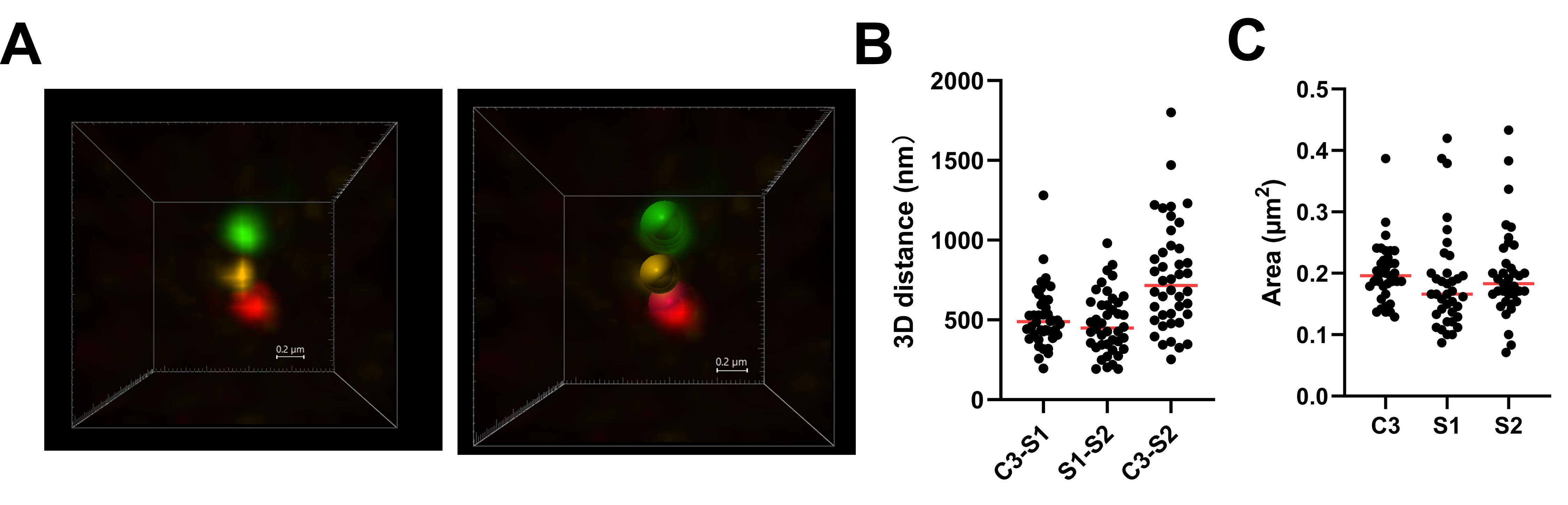

Supplement: S3 Fig — (A) The representative zoom-in images of C3 (green), S1 (brown), and S2 (red) spots. The left is the original processed image and the right is the spot-module mimic image. Scale bars, 0.2 μm. (B) The distribution of 3D distance with C3-S1, S1-S2, and C3-S2. Red lines indicate the mean value. n = 42. (C) The area measurement of C3, S1, and S2 spots. n = 37. The red line indicates the mean area of each group. The underlying data associated with this figure are available in S8 Data. (TIF) [file pbio.3003009.s006.TIF]

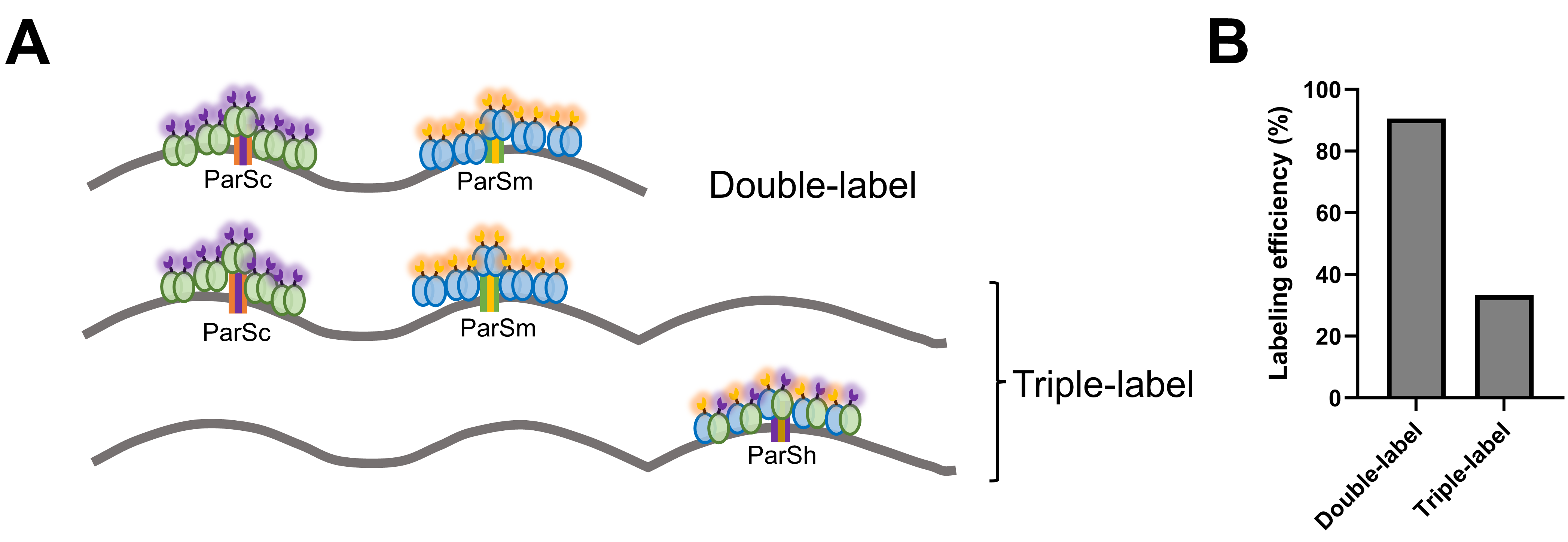

Supplement: S4 Fig — (A) The diagram of double and triple color DNA labeling. (B) Labeling efficiency of double- and triple-color DNA imaging. n = 42 cells for double-label, n = 42 cells for triple-label. The underlying data associated with this figure are available in S9 Data. (TIF) [file pbio.3003009.s007.TIF]

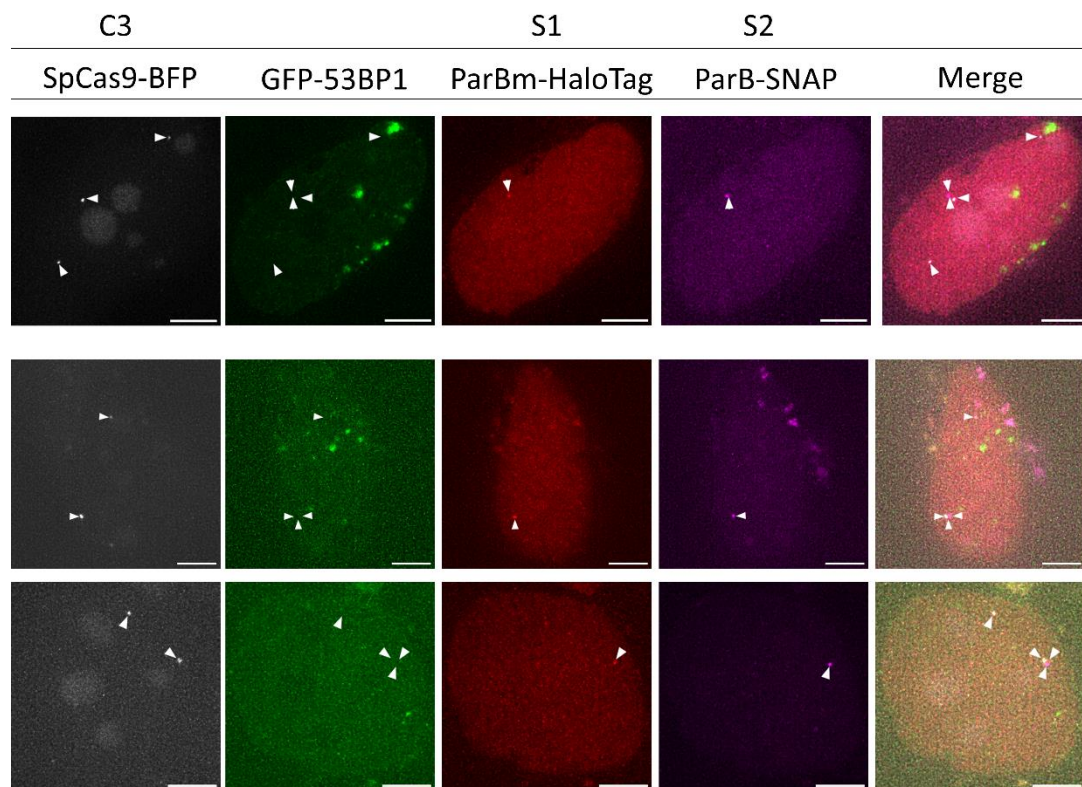

Supplement: S5 Fig — SgRNA-C3, SpCas9-BFP, GFP-53BP1, ParBm-HaloTag, and ParB-SNAP plasmids are transfected together into cells. Arrowheads indicate the positions of C3, S1, and S2 loci. Scale bars, 5 μm. (PDF) [file pbio.3003009.s008.pdf]

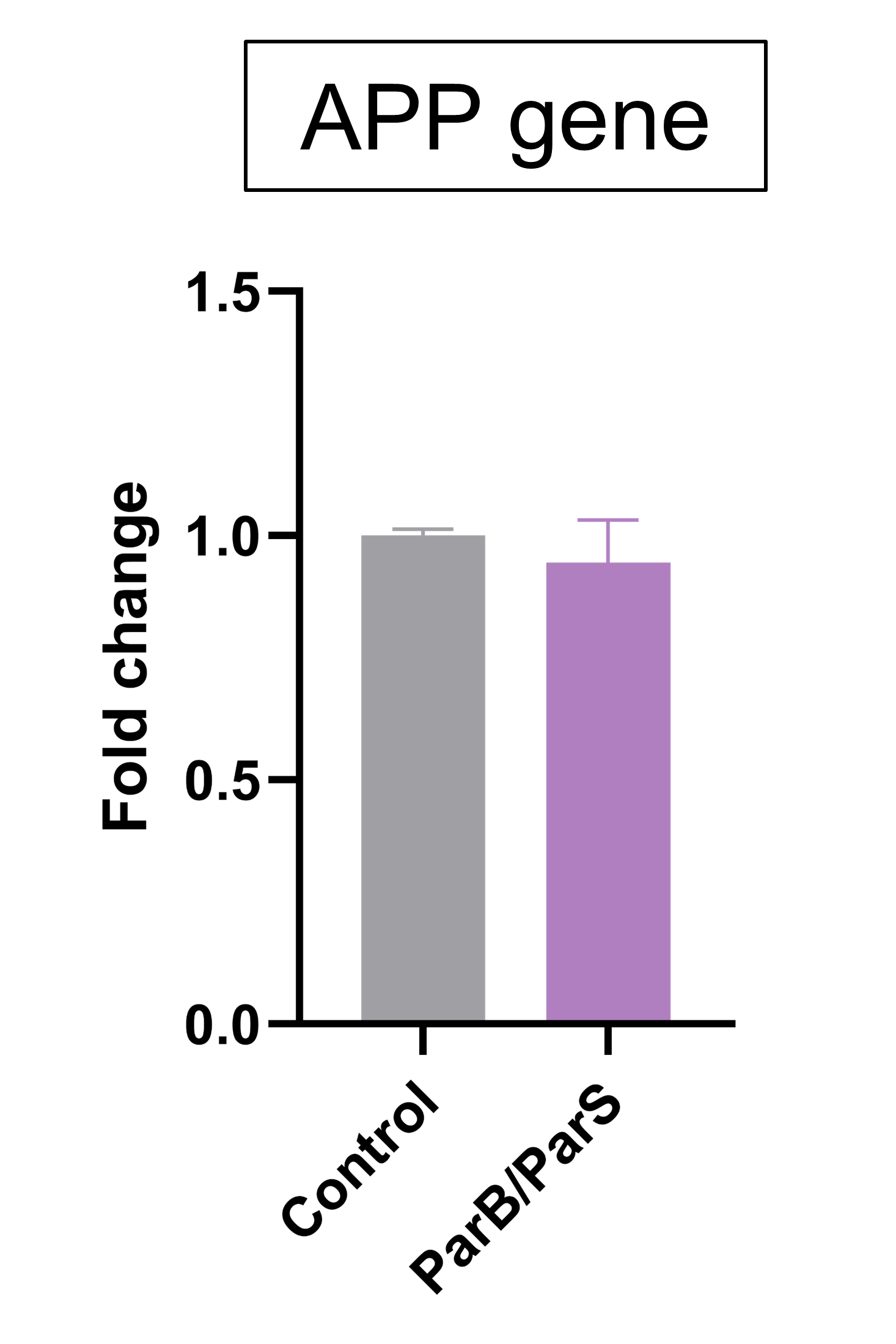

Supplement: S6 Fig — We transfect TtParB plasmid or control plasmid into U2OS-8×ParSc-APP-8×ParSm cells. Total RNA is extracted to complete RT-qPCR testing the expression level of APP gene. The underlying data associated with this figure are available in S10 Data. (TIF) [file pbio.3003009.s009.TIF]

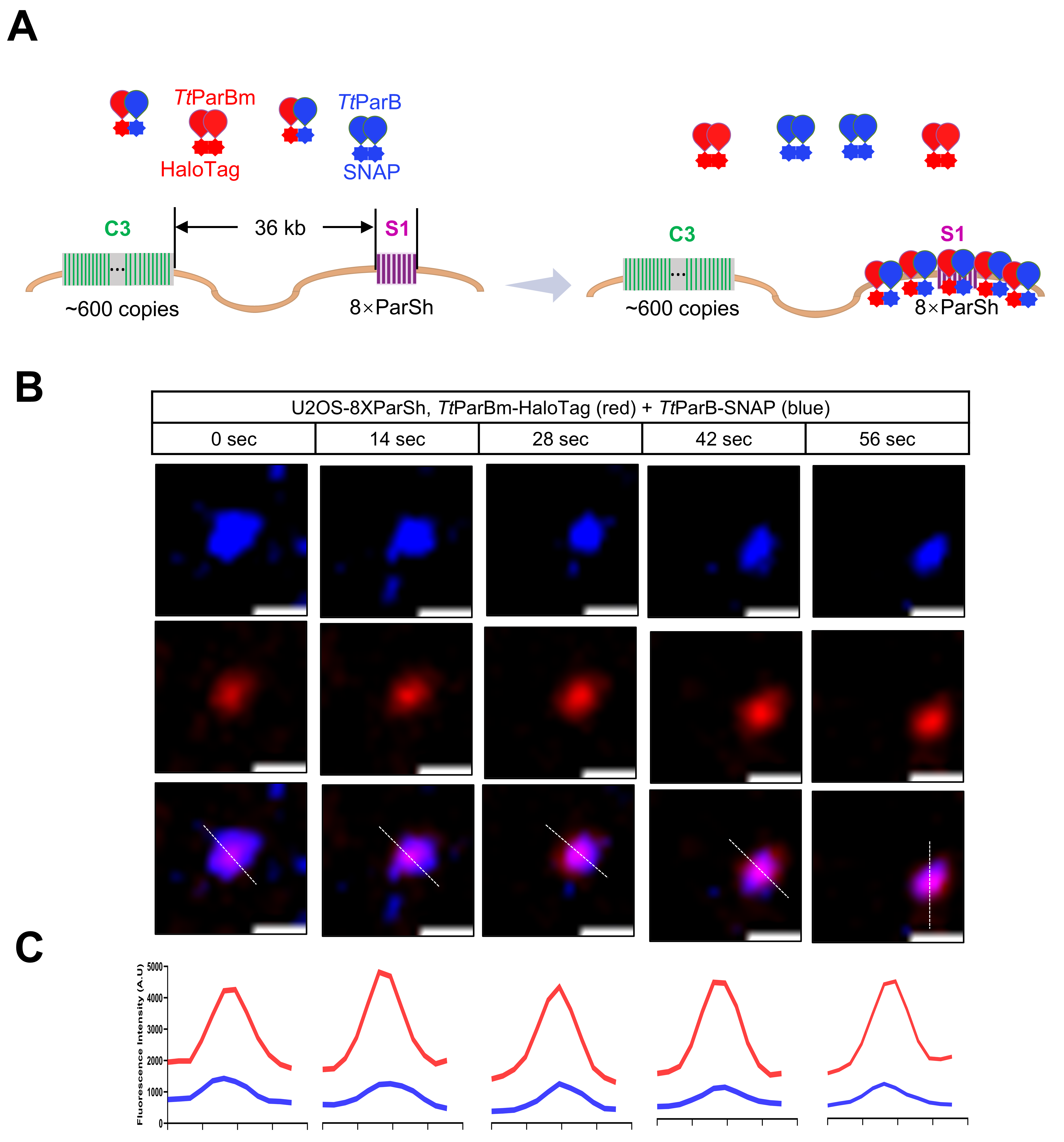

Supplement: S7 Fig — (A) The diagram of labeling the S1 locus by the ParB-ParS hybrid system; 8×ParSh was integrated into the S1 locus and labeled by the heterodimer of TtParB and TtParBm. (B) Time-lapse of the S1 locus labeled by the ParB-ParS hybrid system. Both TtParBm-HaloTag and TtParB-SNAP labeled the S1 locus in the ParB-ParS hybrid system. The S1 locus was tracked over 56 s. Scale bars, 0.5 μm. (C) Plots of the fluorescence intensity of the S1 locus indicated by the dashed lines in B. Red line represents the fluorescence intensity of TtParBm-HaloTag. Blue line represents the fluorescence intensity of TtParB-SNAP. The length of each dashed line is 0.7 μm. (TIF) [file pbio.3003009.s010.TIF]

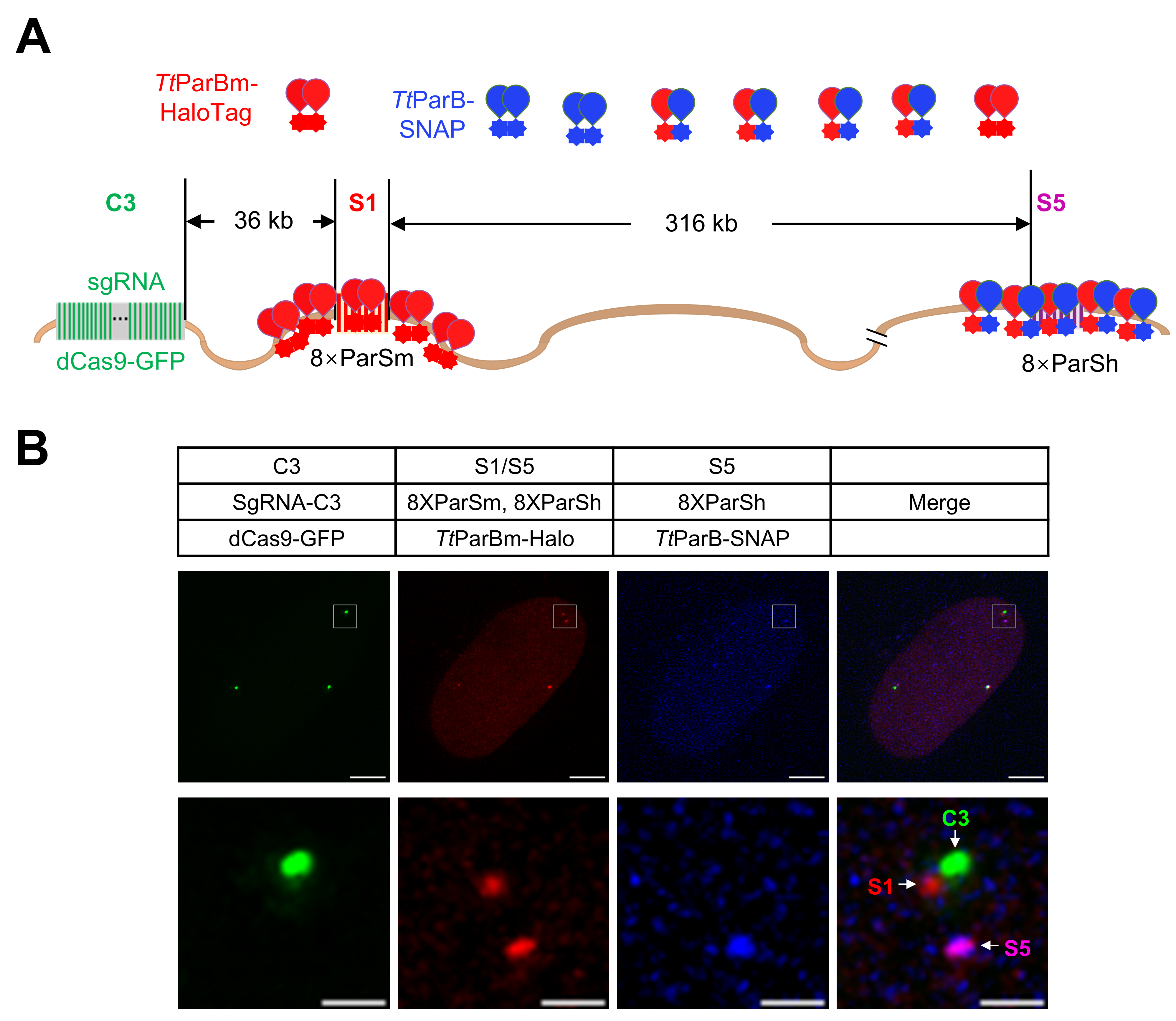

Supplement: S8 Fig — (A) The schematic of labeling S1 and S5 loci by ParSm or ParSh; 8×ParSm and 8×ParSh were integrated into U2OS at the S1 and S5 loci respectively, downstream of C3 repeat on chromosome 3. The dimeric TtParBm-HaloTag and heterodimeric TtParBm-HaloTag/TtParB-SNAP were used for labeling S1 or S5, respectively. (B) Representative images of simultaneous imaging of the S1 and S5 loci. TtParB-SNAP and TtParBm-HaloTag along with dCas9-GFP and sgRNA-C3 for labeling of C3 repeats are transfected into cells to label S1, S5, and C3 repeat loci. The bottom panel shows the simultaneous labeling of C3 (green), S1 (red), and S5 (magenta). Scale bars, 5 μm for images with the whole cells and 1 μm for zoomed images. (TIF) [file pbio.3003009.s011.TIF]

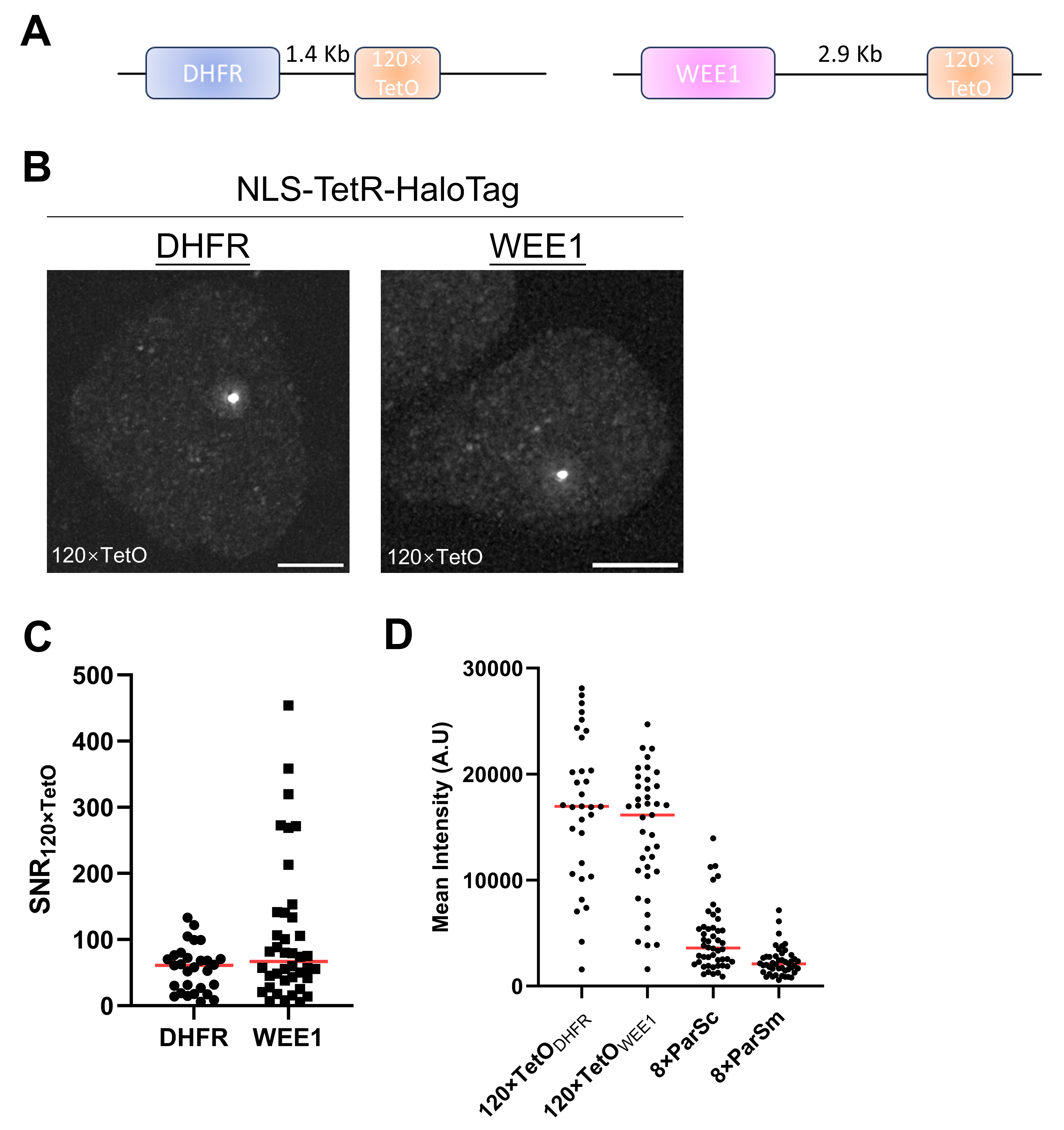

Supplement: S9 Fig — (A) The labeling strategy for DHFR and WEE1 genes by 120-mer TetO array. (B) Representative images of DHFR and WEE1 genes labeling with TetO/TetR system. Scale bars, 5 μm. (C) The SNR of 120-mer TetO/TetR system. n = 29 for DHFR group, and n = 40 for WEE1 group. Red lines indicate mean value. (D) The mean intensity of signal foci for 120-mer TetODHFR/TetR, 120-mer TetOWEE1/TetR, 8-mer ParSc/ParB, and 8-mer ParSm/ParBm systems. n = 32, 39, 48, 46 for each group from left to right. Red line indicates mean value for each group. The underlying data associated with this figure are available in S11 Data. (TIF) [file pbio.3003009.s012.TIF]
